# Race/ethnicity-stratified fine-mapping of the MHC locus reveals genetic variants associated with late-onset asthma

**DOI:** 10.3389/fgene.2023.1173676

**Published:** 2023-06-21

**Authors:** Eunice Y. Lee, Wonson Choi, Adam B. Burkholder, Lalith Perera, Jasmine A. Mack, Frederick W. Miller, Michael B. Fessler, Donald N. Cook, Peer W. F. Karmaus, Hideki Nakano, Stavros Garantziotis, Jennifer H. Madenspacher, John S. House, Farida S. Akhtari, Charles S. Schmitt, David C. Fargo, Janet E. Hall, Alison A. Motsinger-Reif

**Affiliations:** ^1^ Biostatistics and Computational Biology Branch, National Institute of Environmental Health Sciences, Durham, NC, United States; ^2^ Genomics and Bioinformatics Laboratory, Seoul National University, Seoul, Republic of Korea; ^3^ National Institute of Environmental Health Sciences, Durham, NC, United States; ^4^ Genomic Integrity and Structural Biology Laboratory, National Institute of Environmental Health Sciences, Durham, NC, United States; ^5^ Department of Obstetrics and Gynecology, University of Cambridge, Cambridge, United Kingdom; ^6^ Environmental Autoimmunity Group, Clinical Research Branch, National Institute of Environmental Health Sciences, Durham, NC, United States; ^7^ Immunity, Inflammation and Disease Laboratory, National Institute of Environmental Health Sciences, Durham, NC, United States; ^8^ Immunogenetics Group, National Institute of Environmental Health Sciences, Durham, NC, United States; ^9^ Clinical Research Branch, National Institute of Environmental Health Sciences, Durham, NC, United States; ^10^ Division of Translational Toxicology, National Institute of Environmental Health Sciences, Durham, NC, United States

**Keywords:** MHC, HLA allele, immune function, race, ethnicity, late-onset asthma

## Abstract

**Introduction:** Asthma is a chronic disease of the airways that impairs normal breathing. The etiology of asthma is complex and involves multiple factors, including the environment and genetics, especially the distinct genetic architecture associated with ancestry. Compared to early-onset asthma, little is known about genetic predisposition to late-onset asthma. We investigated the race/ethnicity-specific relationship among genetic variants within the major histocompatibility complex (MHC) region and late-onset asthma in a North Carolina-based multiracial cohort of adults.

**Methods:** We stratified all analyses by self-reported race (i.e., White and Black) and adjusted all regression models for age, sex, and ancestry. We conducted association tests within the MHC region and performed fine-mapping analyses conditioned on the race/ethnicity-specific lead variant using whole-genome sequencing (WGS) data. We applied computational methods to infer human leukocyte antigen (HLA) alleles and residues at amino acid positions. We replicated findings in the UK Biobank.

**Results:** The lead signals, rs9265901 on the 5’ end of HLA-B, rs55888430 on HLA-DOB, and rs117953947 on HCG17, were significantly associated with late-onset asthma in all, White, and Black participants, respectively (OR = 1.73, 95%CI: 1.31 to 2.14, *p* = 3.62 × 10^−5^; OR = 3.05, 95%CI: 1.86 to 4.98, *p* = 8.85 × 10^−6^; OR = 19.5, 95%CI: 4.37 to 87.2, *p* = 9.97 × 10^−5^, respectively). For the HLA analysis, HLA-B*40:02 and HLA-DRB1*04:05, HLA-B*40:02, HLA-C*04:01, and HLA-DRB1*04:05, and HLA-DRB1*03:01 and HLA-DQB1 were significantly associated with late-onset asthma in all, White, and Black participants.

**Conclusion:** Multiple genetic variants within the MHC region were significantly associated with late-onset asthma, and the associations were significantly different by race/ethnicity group.

## 1 Introduction

Asthma is a chronic disease of the airways characterized by inflammation, mucus production, and reversible airway obstruction that impairs normal breathing. According to the Centers for Disease Control and American Thoracic Society, 21 million adults in the United States have asthma, and $80 billion is spent annually on healthcare related to the disease (American Thoracic Society, 2018; Centers for Disease Control, 2022). The complex etiology of asthma involves multiple factors, including genetic and environmental risk factors.

Recent studies have revealed that asthma is not a single disease but rather comprises multiple phenotypes (Anderson, 2008; Lötvall et al., 2011). This phenotypic heterogeneity likely stems from the involvement of distinct biological mechanisms, a concept captured by the term “asthma endotypes.” An improved understanding of these endotypes may reveal novel pathways that can be selectively targeted for specific forms of asthma. Our understanding of early-onset, or childhood, asthma has improved, and genetic predisposition is known to play an important role. In general, individuals with early-onset asthma respond well to inhaled corticosteroids, which is the gold standard for asthma treatment (Hirano and Matsunaga, 2018). By contrast, individuals with late-onset asthma are often steroid-resistant, and the potential mechanisms that lead to late-onset asthma remain poorly understood (Hirano and Matsunaga, 2018). In particular, little is known about genetic predisposition to late-onset asthma. Improved knowledge of genes that affect late-onset asthma may reveal novel biologic pathways for targeted therapies.

Genome-wide association studies (GWAS) are a fruitful approach to identifying genes associated with a wide array of diseases, including asthma. GWAS have consistently identified significant associations between various asthma phenotypes and genes within the major histocompatibility complex (MHC) and human leukocyte antigen (HLA) loci (Li et al., 2010; Galanter et al., 2014; Clay et al., 2022). The human MHC region, located on chromosome 6 with an approximate size of 150–180 Mb, is the most gene-dense area of the genome and harbors highly polymorphic genes, including HLA genes. The HLA system encodes a peptide-binding groove of the HLA molecule critical for antigen binding and T cell recognition and the subsequent activation of immune response upon antigen challenge and thus plays an essential role in adaptive immunity. However, the pathological roles of genes within the MHC region and HLA alleles in late-onset asthma remain unclear.

Prior GWAS have sometimes failed to replicate genetic associations in asthma, which has hampered efforts to delineate plausible mechanisms. This failure may be due to population heterogeneity, diverse asthma phenotypes and endotypes, and limitations of single nucleotide polymorphism (SNP)-level studies. In addition, inference of accurate high-resolution HLA alleles and amino acid residues remains challenging due to the tight linkage disequilibrium (LD) across disease-associated MHC haplotypes and the highly polymorphic nature of associated variants. To address these issues and identify putative causal variants, we conducted race/ethnicity-stratified fine-mapping studies of the MHC region for late-onset asthma utilizing WGS samples collected from a North Carolina-based multiethnic adult cohort. We also applied appropriate and high-accuracy computational methods/tools to infer HLA alleles and amino acid positions and residues for individuals of African and European ancestry using WGS data. The results show heterogeneity at three levels of resolution, namely, single nucleotide variants (SNVs), HLA alleles, and amino acids.

## 2 Materials and methods

### 2.1 Study participants: PEGS cohort

The ongoing North Carolina-based Personalized Environment and Genes Study (PEGS) (*N* = 19,672) began recruiting participants in 2002 and collects questionnaire-based exposome and health history data. The race/ethnicity of PEGS participants is categorized into American Indian/Alaska Native, Asian, Black, Hispanic, Native Hawaiian/Pacific Islander, White, and other/multiple races/ethnicities. However, due to small sample sizes, we focused our analyses on three racial/ethnic groups: non-Hispanic Black individuals, non-Hispanic White individuals, and all individuals. PEGS is described in detail elsewhere (Lee et al., 2022). Local institutional review boards approved the studies, and all participants provided written informed consent.

### 2.2 Asthma case and control definition

For the late-onset asthma outcome, we assigned participants to the case group if they answered “YES” when asked if they have ever been diagnosed with asthma (as defined by Global Initiative for Asthma criteria) (Global Initiative for Asthma, 2022) by a physician, currently have asthma, do not have other respiratory diseases, including idiopathic pulmonary fibrosis (IPF), chronic obstructive pulmonary disease (COPD), and tuberculosis (TB), and were diagnosed with asthma at the age of 20 years or more. We assigned participants to the control group if they answered “NO” when asked if they have ever been diagnosed with asthma, have other respiratory diseases, including IPF, COPD, and TB, and have a regular cough and/or breathlessness, regular wheezing, or whistling in the chest.

### 2.3 MHC region-wide allelic association tests

We performed race/ethnicity-stratified MHC region-wide association testing for late-onset asthma in 3,641 PEGS participants, adjusting for age, sex, and four ancestry groups. The results of the MHC region-wide association tests for each ancestry are described below and summarized in the Supplementary Material.

### 2.4 Fine-mapping: conditional analysis

We built a multi-SNP logistic regression model, applying a forward selection procedure for all, White, and Black participants and adjusted for age, sex, and four ancestry groups, conditional on the lead SNP (Lee et al., 2020). Details of the fine-mapping conditional analysis can be found in the Supplementary Material (Supplementary Section S1.5; Supplementary Table S1).

### 2.5 Validation

To validate our results from the association tests, we examined associations of the lead SNPs for each analysis (all, White, and Black participants) in GWAS results from the Pan-UK Biobank (Pan-UK Biobank, 2022) for asthma and asthma-related phenotypes. The UK Biobank (RRID:SCR_012815) is a large-scale biomedical database with both genetic and phenotype data for several common diseases and traits for approximately 500,000 participants from the United Kingdom (Sudlow et al., 2015). The Pan-UK Biobank study conducted a multi-ancestry GWAS of 7,228 phenotypes, including asthma and asthma-related phenotypes such as allergic rhinitis, lung function, and respiratory infections. Summary statistics are available for each ancestry group and a meta-analysis across all populations.

### 2.6 Functional annotation of genetic variants

We conducted annotation for the lead SNP and candidate causal SNPs identified from the conditional analyses using publicly available data (Boyle et al., 2012; Carithers et al., 2015; Martin et al., 2017; Carvalho-Silva et al., 2019; Luo et al., 2020). The Supplementary Material provides more information (Supplementary Section S1.6).

### 2.7 Assembly of HLA alleles: Kourami

We used Kourami (RRID:SCR_022280), a graph-guided assembly technique, to generate four- and six-digit HLA alleles for six common HLA genes types (HLA-A, -B, -C, -DQA, DQB, and -DRB) and 14 additional HLA genes (HLA-DOA, -DOB, -DMA, -DMB, -DPA1, -DPB1, -DRQ, -DRB3, -DRB5, -F, -G, -H, -J, and -L) (Lee and Kingsford, 2018). The Supplementary Material provides more information on Kourami (Supplementary Section S1.7).

### 2.8 Amino acid position inference

In addition to HLA alleles, we inferred residues at amino acid positions. We applied the CookHLA and HLA analysis toolkit (HATK) enrichment-free computational HLA imputation/inference methods (Choi et al., 2021; Cook et al., 2021). The Supplementary Material provides details (Supplementary Section S1.8).

### 2.9 Structure implications

We selected the highest-resolution X-ray crystal structures for HLA-DRB (pdb ID: 4x5w, resolution 1.34A), HLA-B (pdb ID: 1K5N, resolution 1.09 A), and HLA-C (pdb ID: 6JTO, resolution 1.70A). Structures were rendered using Chimera (RRID:SCR_002959).

We conducted all statistical analyses in R Project for Statistical Computing (RRID:SCR_001905, version 4.2.1) and PLINK 2.0 (RRID:SCR_001757) (Purcell et al., 2007; Habu et al., 2014).

## 3 Results

### 3.1 Study participants

**FIGURE 1:**
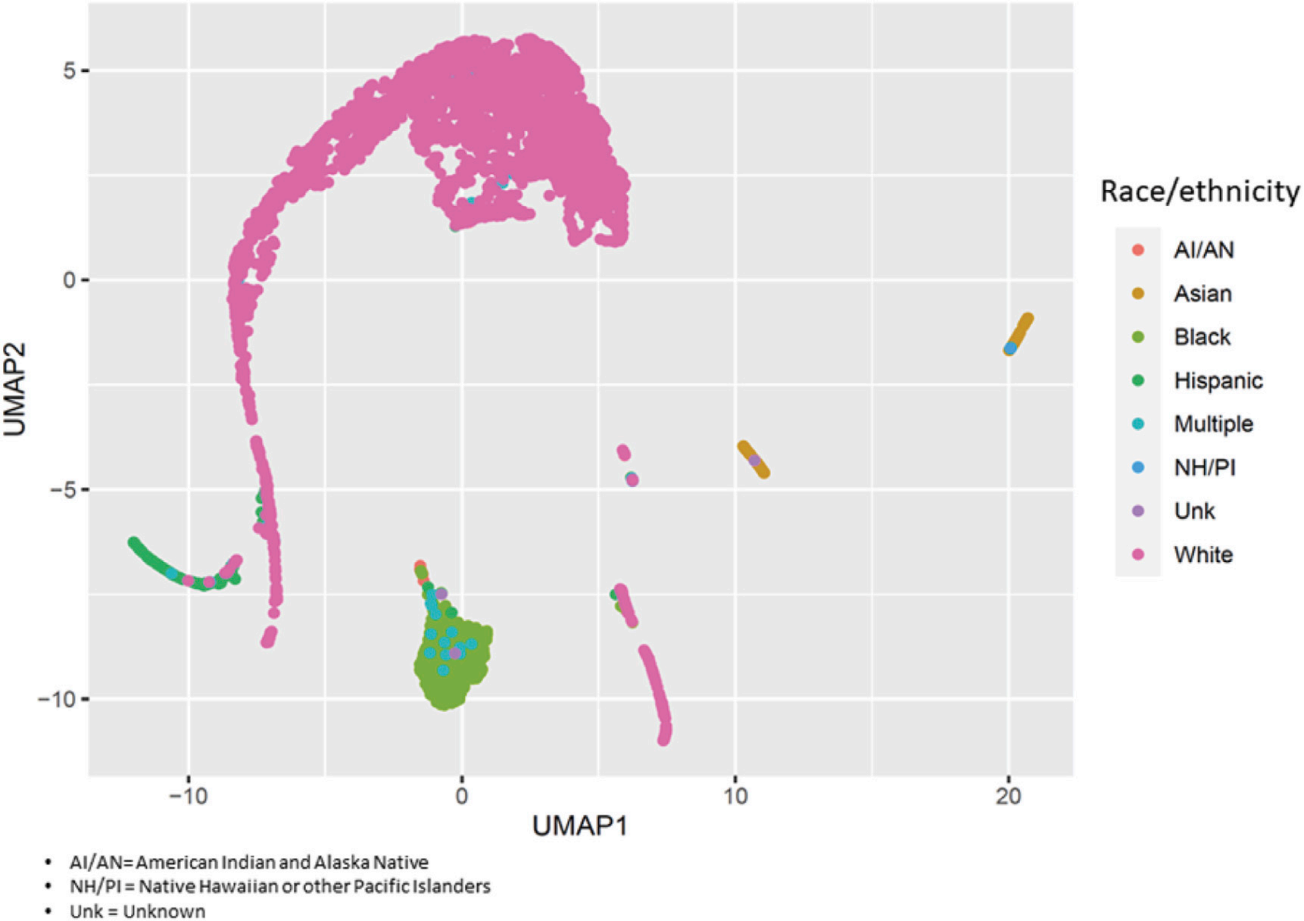
Population structure of PEGS participants. We applied uniform manifold approximation and projection (UMAP) to visualize the genetic variability of study participants using the first 40 principal components of whole-genome sequencing data. Each circle represents a study participant, and the colors represent their self-reported race/ethnicity.

On average, asthma cases were older than controls (54.3 ± 11.1 and 50.3 ± 14.8 years, respectively: *p* = 4.1 × 10^−4^) (Supplementary Table S2). The proportion of males in the case group was lower than in the control group (15.5% vs. 32.8%: *p* = 2.8 × 10^−6^). The small differences in genomic ancestry proportions between cases and controls were not statistically significant except for Black participants (18.5% ± 32.8% and 12.6% ± 28.4%, respectively: *p* = 7.3 × 10^−3^). Figure 1 displays the variability of genomic ancestry proportions for individual participants, and Supplementary Figure S2 outlines the population structure of PEGS participants by self-reported race and ethnicity.

### 3.2 MHC region-wide association studies

**FIGURE 2:**
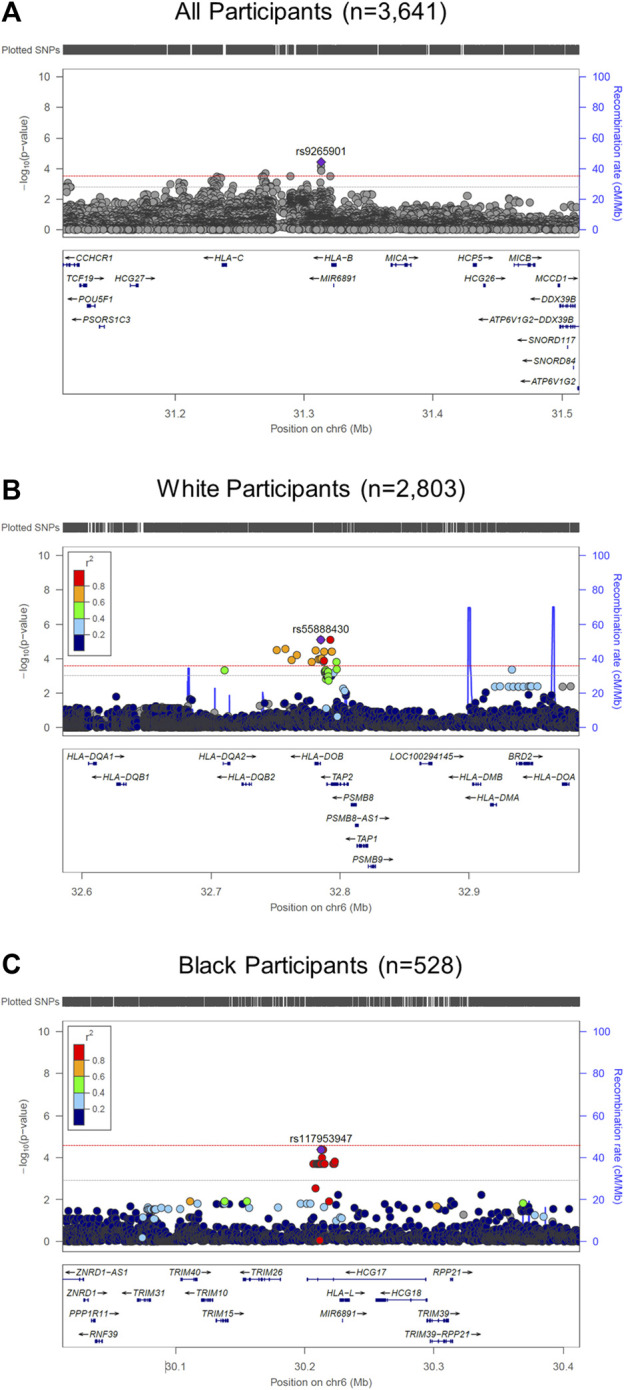
LocusZoom (RRID:SCR_009257) plots of MHC region-wide studies. **(A)** For all participants (*n* = 3,641), rs9265901 (overlapping gene: HLA-B) was associated with late-onset asthma; **(B)** For White participants, rs55888430, an intron variant of HLA-DOB, was significantly associated with late-onset asthma; **(C)** For Black participants, rs117953947, which overlaps with HCG17, was significantly associated with late-onset asthma. We adjusted all regression models for age, sex, and four ancestries. The red and gray dotted lines represent the significance and suggestive thresholds.

**FIGURE 3:**
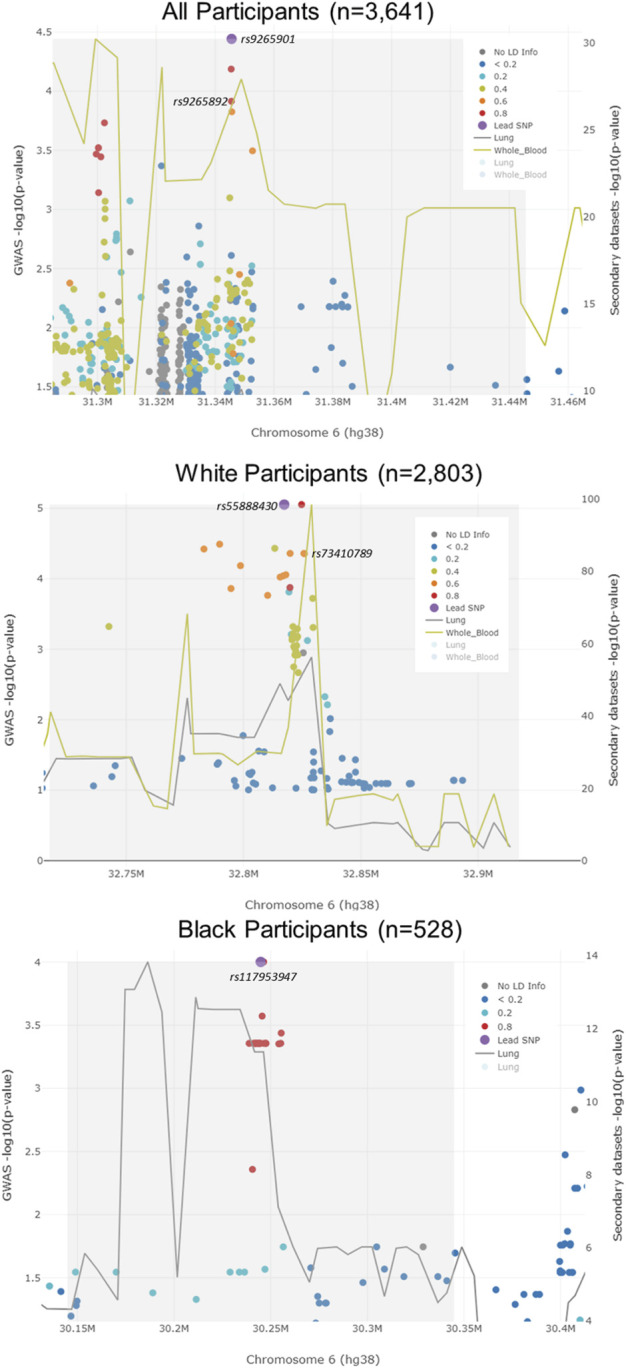
LocusFocus plots of colocalization analysis results. The lead SNP for all participants (rs9265901) was not associated with the expression of HLA-B in lung tissue or whole blood. However, an SNP that was highly correlated with the lead SNP (rs9265892) was associated with the expression of HLA-B in whole blood. The lead SNP (rs55888430) for White participants was not associated with the expression of HLA-DOB. An LD SNP for the lead SNP (rs73410789) was associated with HLA-DOB expression in whole blood. The lead SNP for Black participants, rs117953947, was not associated with the expression of HCG17 in lung tissue.

**FIGURE 4:**
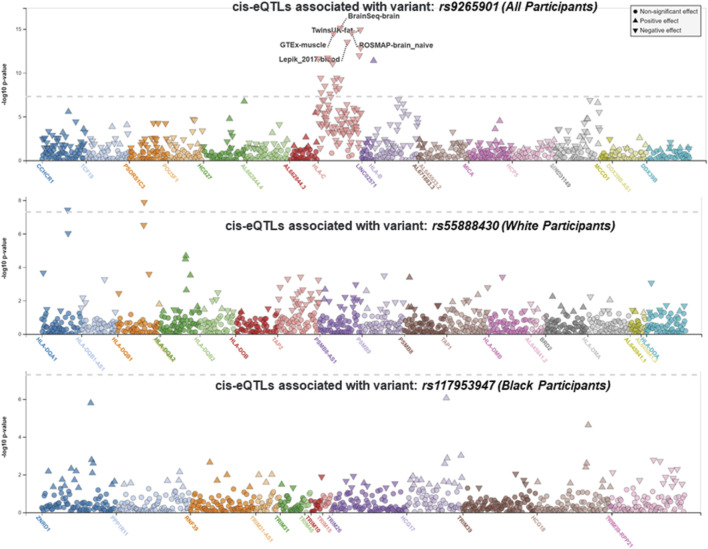
LocusZoom (RRID:SCR_009257) plots displaying cis-eQTLs. The lead SNPs for all participants (rs9265901), White participants (rs55888430), and Black participants (rs117953947) were weakly correlated with cis-eQTLs for multiple genes in multiple tissues (from top to bottom, respectively). The gray dotted line represents the significance threshold.

We performed race/ethnicity-stratified MHC region-wide association testing for late-onset asthma in 3,641 PEGS participants, adjusting for covariates. We discovered two MHC region-wide peaks for all and White participants and a suggestive peak for Black participants after multiple testing correction (Figure 2). The most significant association for all participants is located near *HLA-B*. The lead signal, rs9265901, is on the 5’ end of *HLA-B* (Figure 3). A one-unit increase in the affected allele (minor allele: G) was associated with 67% increased odds of being a case (OR = 1.73, 95%CI: 1.31 to 2.14, *p* = 3.62 × 10^−5^). For White participants, the lead SNP, rs55888430, is on *HLA-DOB* (OR = 3.05, 95%CI: 1.86 to 4.98, *p* = 8.85 × 10^−6^). For Black participants, the lead SNP, rs117953947, is on *HCG17* (OR = 19.5, 95%CI: 4.37 to 87.2, *p* = 9.97 × 10^−5^). The lead SNPs for each reference ancestry are weakly to moderately correlated with cis-eQTLs for multiple genes in multiple tissues according to the GTEx eQTL Browser (RRID:SCR_001618) (Table 1; Figure 4) (Carithers et al., 2015). The results of the MHC region-wide association tests for each ancestry are summarized in the Supplementary Material.

**TABLE 1:** Amino acids analysis results for all, White, and Black participants. HLA amino acid positions with a particular residue were associated with late-onset asthma. We adjusted all regression models for age, sex, and four ancestries.

HLA amino acids analysis						
All participants						
HLA-(gene)	AA position	Genetic position	Residue	OR (95% CI)		*p*-value
*C*	90	31347357_exon2	A/D	1.40 (1.13 to 1.74)		2.34 × 10^−3ǂ^
*C*	73	31347408_exon2	T/A	1.44 (1.16 to 1.79)		9.90 × 10^−4ǂ^
*DRB1*	38	32660026_exon2	L	2.59 (1.56 to 4.28)		2.16 × 10^−4ǂ^
*DRB1*	38	32660026_exon2	V	2.04 (1.26 to 3.30)		3.64 × 10^−3ǂ^
*DRB1*	37	32660029_exon2	L	2.59 (1.56 to 4.28)		2.16 × 10^−4ǂ^
White participants						
*B*	103	31432162_exon3	L/V	0.48 (0.30 to 0.74)		1.08 × 10^−3§^
Black participants						
*C*	73	31347408_exon2	T/A	2.26 (1.38 to 3.72)		1.31 × 10^−3ʄ^

Amino acid position analysis: ^ǂ^Pooled ancestry: significant *p*-value = 3.9 × 10^−4^; suggestive *p*-value = 3.9 × 10^−3^. ^§^EUR ancestry: significant *p*-value = 5.6 × 10^−4^; suggestive *p*-value = 5.6 × 10^−3^. ^ʄ^AFR ancestry: significant *p*-value = 3.4 × 10^−4^; suggestive *p*-value = 3.4 × 10^−3^.

### 3.3 Fine-mapping: conditional analysis

To identify candidate causal variants, we conducted conditional analyses to determine associations between late-onset asthma and the affected/minor allele at an SNP. Because the different allelic association signals observed for different ancestries were not due to a single SNP, we further investigated the joint effects of multiple SNPs on late-onset asthma.

Using multi-SNP logistic regression models with a forward selection procedure, we identified 10, 9, and 12 significant, independent SNPs for all, White, and Black participants, respectively (Table 2). The effect estimates in Table 2 represent the association between an SNP and late-onset asthma, conditioned on the lead SNP and adjusted for covariates and additional SNPs. The table contains only SNPs that remained significant after adjusting for additional SNPs and covariates. Table 3 displays the associations and minor allele frequencies (MAF) of the lead SNPs across all strata. The Supplementary Material provides a full list of the selected SNPs (Supplementary Table S3).

**TABLE 2:** Fine-mapping of the MHC region harboring race/ethnicity-specific signals using sequenced data. We evaluated the joint effects of multiple variants on late-onset asthma with stepwise regression models. Conditional analyses of race/ethnicity-specific lead SNPs identified potential causal SNPs in the MHC region for all, White, and Black participants.

All participants						
Variants	Position	Gene	Alleles	Freq	OR (95% CI)^a^	*p*-value
*rs9265901*	6:31345615	HLA-B	A|G		1.01 (1.00, 1.03)	0.05
*rs59377618*	6:32820360	HLA-DOB	T|C		1.02 (1.01, 1.04)	4.42 × 10^−3^
*6:33097139*	6:33097139	HLA-DPA1	G|A		0.98 (0.97, 0.99)	4.62 × 10^−3^
*rs2894334*	6:33330567	SMIM40	A|C		0.99 (0.98, 0.99)	0.01
*rs5009448*	6:29972711	HLA-A	T|C		1.01 (1.00, 1.02)	0.02
*rs7767589*	6:30402196	RPP21	C|T		1.07 (1.02, 1.13)	7.00 × 10^−3^
*rs73728546*	6:31380112	MICA	C|T		1.05 (1.02, 1.09)	5.24 × 10^−3^
*rs4569*	6:31670030	LY6G5B	C|T		0.98 (0.97, 0.99)	3.21 × 10^−3^
*6:33061419*	6:33061419	HLA-DPB1	C|T		1.10 (1.05, 1.16)	2.23 × 10^−4^
*rs9268541*	6:32416750	BTNL2	T|C		1.03 (1.00, 1.05)	0.02
*rs62407970*	6:32969217	HLA-DMA	G|A		1.03 (1.01, 1.05)	6.89 × 10^−3^
White participants						
*rs55888430*	6:32817365	HLA-DOB	G|A		1.03 (0.99, 1.07)	0.06
*rs5009448*	6:29972711	HLA-A	T|C		1.02 (1.01, 1.03)	1.65 × 10^−3^
*rs116062523*	6:31383028	MICA	T|C		1.08 (1.03, 1.14)	3.64 × 10^−3^
*rs7754362*	6:33143251	HCG24, COL11A2	A|C		0.98 (0.97, 0.99)	2.95 × 10^−4^
*6:29800571*	6:29800571		A|C		1.07 (1.01, 1.13)	0.02
*6:29816791*	6:29816791		A|T		1.05 (1.00, 1.10)	0.04
*6:31932537*	6:31932537	CFB	T|C		1.07 (1.04, 1.11)	2.91 × 10^−5^
*rs1632859*	6:31002121	MUC22	G|A		1.01 (1.00, 1.03)	0.04
*rs17208209*	6:32227599	NOTCH4	G|A		1.04 (1.00, 1.08)	0.04
*rs115337486*	6:33387741	KIFC1	C|T		1.04 (1.00, 1.08)	0.04
Black participants						
*rs117953947*	6:30244862	TRIM26, HCG17	G|A		1.13 (0.98, 1.30)	0.09
*rs150891754*	6:28608725	ZBED9	A|G		1.27 (1.13, 1.44)	1.20 × 10^−4^
*rs17200414*	6:31449359	MICB	C|T		1.21 (1.05, 1.38)	7.90 × 10^−3^
*rs200535118*	6:31352792	HLA-B	G|T		1.19 (1.01, 1.40)	0.04
*rs62408569*	6:29406052	OR5V1, OR12D2	C|T		1.31 (1.13, 1.52)	4.04 × 10^−4^
*rs181714996*	6:33331782	SMIM40	T|C		1.19 (1.07, 1.32)	1.05 × 10^−3^
*rs115571466*	6:32998090	HLA-DOA	A|G		1.20 (1.07, 1.35)	1.71 × 10^−3^
*rs4713423*	6:31034524	MUC22	G|C		1.04 (1.00, 1.08)	0.03
*rs17198965*	6:31308446	HLA-C	C|G		1.08 (1.02, 1.16)	0.01
*rs73403122*	6:32501344	HLA-DRB5	A|G		1.21 (1.07, 1.36)	1.92 × 10^−3^
*rs17422797*	6:32297751	NOTCH4	C|T		1.26 (1.09, 1.44)	1.38 × 10^−3^
*rs79735834*	6:29517075	MAS1L	A|G		1.08 (1.02, 1.15)	0.01
*rs187569734*	6:31044172	MUC22	G|A		1.28 (1.11, 1.48)	6.82 × 10^−4^

^a^ Odds ratios represent the association between lead SNPs (*rs9265901*, *rs55888430*, *rs117953947*) and late-onset asthma, adjusted for covariates and additional SNPs in forward stepwise regression analyses. We adjusted all regression models for age, sex, and four ancestries.

Note: A full list of the variants identified from conditional analyses is available in the Supplementary Table S4.

**TABLE 3:** Associations and minor allele frequencies (MAF) of the leads SNPs across all strata.

			Pooled		EUR		AFR	
Lead SNPs	MAF	MAF	OR^a^	*p*-value	OR^a^	*p*-value	OR^a^	*p*-value
*rs9265901*	0.15 (G)	EUR: 21%	1.31	3.62 × 10^−5^	1.66	6.91 × 10^−4^	1.79	0.03
		AFR: 15%						
*rs55888430*	0.03 (A)	EUR: 4%	2.24	2.84 × 10^−4^	3.05	8.85 × 10^−6^	0.32	0.26
		AFR: 5%						
*rs117953947*	0.04 (A)	EUR: 2%	1.14	0.76	0.46	0.30	19.5	9.97 × 10^−5^
		AFR: 1%						

^a^ Odds ratios represent the association between lead SNPs (*rs9265901*, *rs55888430*, *rs117953947*) and late-onset asthma, adjusted for covariates and additional SNPs in forward stepwise regression analyses. We adjusted all regression models for age, sex, and four ancestries.

After identifying candidate causal variants from the conditional analyses, we performed functional annotation of these SNPs to elucidate their potential biological roles in late-onset asthma in PEGS participants. Supplementary Table S4 is a full list of candidate causal variants. Based on annotation from publicly available data, three SNPs identified for all participants exhibit a regulatory function in white blood cells and lung tissue (Supplementary Table S5). rs4569, a 3′UTR variant of *LY6G5B*, was strongly correlated with the negative expression of *LY6G5C* in whole blood and lung tissue (*p* = 1.3 × 10^−119^ and *p* = 1.8 × 10^−43^, respectively) (Supplementary Table S4). A 5′UTR variant of *BRD2*, rs62407970, intersected with strong promoter-like signatures, including a DNase I hypersensitive site and a histone modification (H3K4me3 and H3K27ac ChIP-Seq) (cCRE accession ID: EH38E3701714). Multiple experimental data indicate that rs62407970 falls within the *POLR2A* transcription factor binding site and may interact with *HLA-DPB2* in human lung fibroblast cells (IMR90) and mesenchymal stem cells (MES) (Boyle et al., 2012).

For White participants, the non-coding exon variant of *HCG24*, rs7754362, intersected with low DNase-seq and H3K27ac ChIP-Seq peaks in lung tissue and CD14^+^ monocytes and high CTCF signals in human lung fibroblast cells (cCRE accession ID: EH38E3701814). In addition, ChIP-Seq experiments indicate that rs7754362 is located within a CTCF transcription factor binding site in blood and lung tissue. Long-range chromatic interaction data suggest that rs7754362 may interact with *HLA-DMA* and *BRD2* in IMR90 and MES cell lines. Within the genomic region identified for Black participants, the intron variant rs17198965 overlapped with strong promoter-like signatures, including high DNase-seq and H3K4me3 ChIP-Seq peaks. Experimental data suggest that rs17198965 resides in the binding site of transcription factor *EZH2* (cCRE accession ID: EH38E3700474).

### 3.4 Validation of lead SNPs

We examined the associations of the lead SNPs from our association analyses with asthma and asthma-related phenotypes in the UK Biobank data using Pan-UK Biobank GWAS results (Pan-UK Biobank, 2022). We found that the lead SNP for all participants, *rs9265901*, is associated with asthma (OR = 1.04, *p* = 3.36 × 10^−4^), atopic dermatitis (OR = 1.12, *p* = 4.33 × 10^−5^), decreased lung function (βeta = −0.01, *p* = 1.47 × 10^−5^), and allergic rhinitis (OR = 1.04, *p* = 5.62 × 10^−6^) in the UK Biobank (Raj et al., 2014). In addition, *rs9265901* is associated with increased lymphocytes (βeta = 0.06, *p* = 2.35 × 10^−135^), eosinophils (βeta = 0.03, *p* = 1.68 × 10^−25^), and neutrophils (βeta = 0.06, *p* = 1.33 × 10^−103^) (Raj et al., 2014). The top SNP for White participants, *rs55888430*, is associated with increased odds of being an asthma case (OR = 1.14, *p* = 1.78 × 10^−4^), having upper respiratory infections (OR = 1.30, *p* = 1.95 × 10^−3^), and having increased eosinophils (βeta = 0.05, *p* = 9.82 × 10^−16^) (Poplin et al., 2018). However, the association of this variant with reduced odds of both late- and early-onset asthma has been reported elsewhere (OR = 0.39, *p* = 3.2 × 10^−3^; OR = 0.41, *p* = 2.0 × 10^−4^, respectively) (Poplin et al., 2018). Lastly, the lead variant for Black participants, *rs117953647*, is associated with opportunistic respiratory infection (OR = 1.71, *p* = 1.82 × 10^−4^), pneumonia (OR = 2.55, *p* = 2.02 × 10^−3^), and increased lymphocytes (βeta = 0.03, *p* = 2.15 × 10^−4^) (Manichaikul et al., 2010), further validating our findings and providing evidence supportive of the proposed ancestry-specific pathways.

### 3.5 HLA analysis: HLA alleles

For all participants, several classical HLA alleles were significantly associated with late-onset asthma after adjusting for covariates (Table 4). The strongest association was HLA-B*40:02 (OR = 3.18, 95%CI: 1.63 to 6.18), which is concordant with the MHC-wide association test result indicating the lead SNP (rs9265901) is located near *HLA-B* (Supplementary Figure S3). The second strongest association was HLA-DRB1*04:05 (OR = 6.94, 95%CI: 2.15 to 22.44). For White participants, two MHC class I alleles, HLA-B*40:02 and HLA-C*04:01, and one MHC class II allele, HLA-DRB1*04:05, were significantly associated with late-onset asthma. HLA-C*04:01 was protective for being a late-onset asthma case (OR = 0.25, 95%CI: 0.09 to 0.70) whereas HLA-B*40:02 and HLA-DRB1*04:05 increased the odds of being a case [(OR = 3.77, 95%CI: 1.91 to 7.44; OR = 8.19, 95%CI: 2.51 to 26.79), respectively]. For Black participants, two MHC class II alleles, HLA-DRB1*03:01 and HLA-DQB1*02:01, were significantly associated with increased risk of being a late-onset asthma case [(OR = 4.00, 95%CI: 1.46 to 10.91; OR = 2.25, 95%CI: 1.09 to 4.65), respectively].

**TABLE 4:** HLA allele analysis results for all, White, and Black participants. Classical HLA alleles of class I and II genes in four-digit resolution were associated with increased and reduced odds of being a late-onset asthma case. We adjusted all regression models for age, sex, and four ancestries.

	Classical HLA allele analysis (4-digit)						
	All participants						
*HLA*-(gene)		* allele:protein	OR (95% CI)^a^	*p*-value	*n*	Freq^b^	Freq
*HLA-B*		*40:02	3.18 (1.63 to 6.18)	6.69 × 10^−4ǂ^	95	0.03	0.02^c^
*HLA-DRB1*		*04:05	6.94 (2.15 to 22.44)	1.22 × 10^−3ǂ^	18	0.005	0.0035^c^
White participants							
*HLA-B*		*40:02	3.77 (1.91 to 7.44)	1.28 × 10^−4§^	82	0.03	0.02^c^
*HLA-C*		*04:01	0.25 (0.09 to 0.70)	7.97 × 10^−3§^	325	0.12	0.13^c^
*HLA-DRB1*		*04:05	8.19 (2.51 to 26.79)	5.03 × 10^−4§^	15	0.005	0.0035^c^
Black participants							
*HLA-DRB1*		*03:01	4.00 (1.46 to 10.91)	6.71 × 10^−3ʄ^	29	0.05	0.07^d^
*HLA-DQB1*		*02:01	2.25 (1.09 to 4.65)	2.91 × 10^−2ʄ^	132	0.25	0.22^d^

^a^ Bi-allelic (presence or absence of the two given residues).

^b^ HLA allele frequency computed from PEGS participants.

^c^ HLA allele frequency computed from the United States population-Caucasian (*n* = 61,655)^35^.

^d^ HLA allele frequency computed from the United States population-African American (*n* = 2,411)^35^.

HLA analysis: ^ǂ^Pooled ancestry: significant *p*-value = 3.3 × 10^−4^; suggestive *p*-value = 3.3 × 10^−3^. ^§^EUR ancestry: significant *p*-value = 2.0 × 10^−3^; suggestive *p*-value = 2.0 × 10^−2^. ^ʄ^AFR ancestry: significant *p*-value = 3.0 × 10^−3^; suggestive *p*-value = 3.0 × 10^−2^.

### 3.6 HLA analysis

In accordance with the HLA allele analysis results, within *HLA-DRB1*, the amino acid variants at positions 37 and 38 were significantly associated with late-onset asthma. Among the three allelic variants at position 38 (leucine, valine, alanine) and five variants at position 37 (serine, phenylalanine, tyrosine, asparagine, leucine), leucine was the strongest risk residue at both positions (Supplementary Figure S4E). Within *HLA-C*, threonine residue at position 73 and alanine residue at position 90 were associated with increased odds of being an asthma case (OR = 1.40, 95%CI: 1.13 to 1.74; OR = 1.44, 95%CI: 1.16 to 1.79, respectively). For White participants, the leucine allele at amino acid 103 within *HLA-B* was associated with reduced odds of being a late-onset asthma case (OR = 0.48, 95%CI: 0.30 to 0.74). For Black participants, threonine residue at position 73 within *HLA-C* was associated with a 126% increase in the odds of being an asthma case.

**FIGURE 5:**
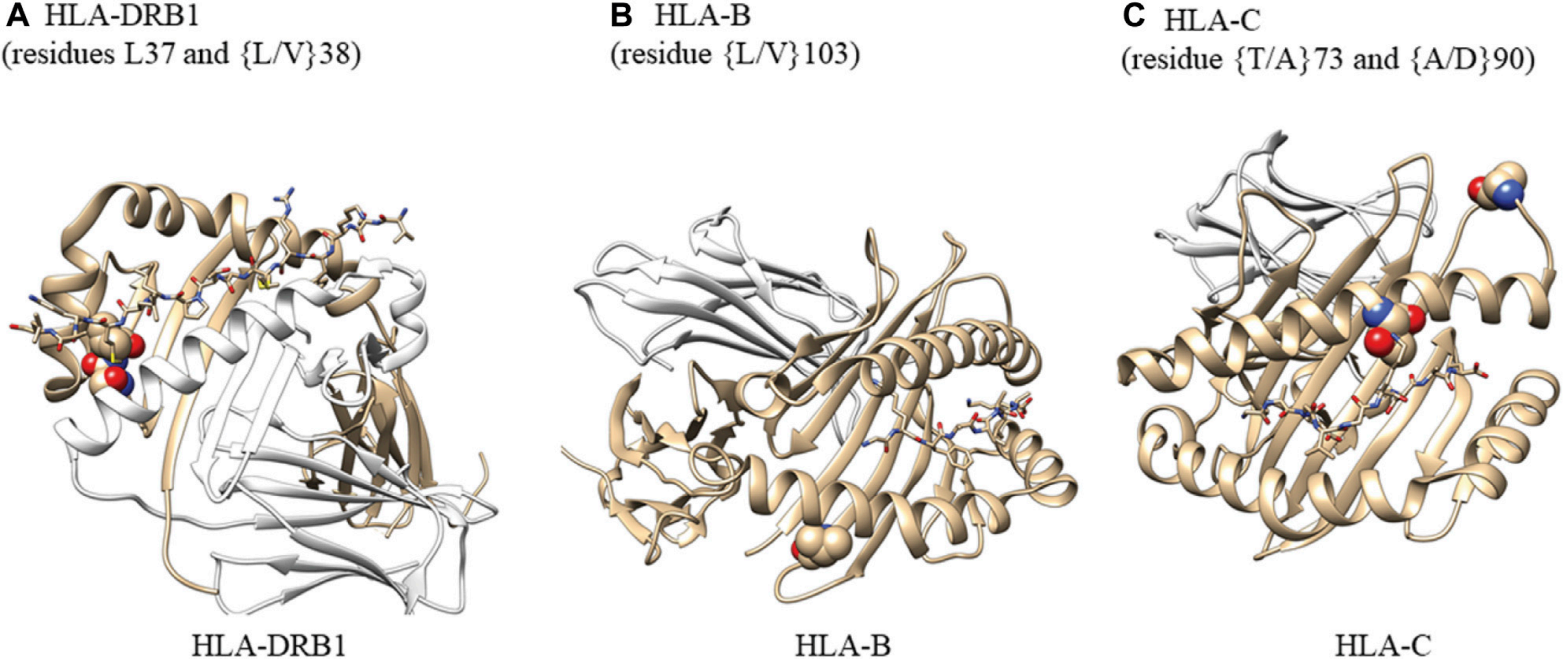
Analysis of amino acid residues showing their potential ability to bind antigens. **(A)** Atoms in residues 37 and 38 are shown as spheres. HLA-DRA (cyan), HLA-DRB1 (gray), M141 TCR𝜶 (wheat), and M141 TCRβ (yellow) are shown as ribbons. Residues 37 and 38 are located on the peptide-binding surface. One of the two residues is also in contact with a helix from HLA-DRA that comprises the complex. We created this figure using PDB ID (4x5w); **(B)** Atoms in residues 103 are shown as spheres. HLA-B (gray) and β-2-macroglobulin (wheat) are shown as ribbons. The small peptide-binding pocket (lines) is composed of three helices, one of which directly interacts with residue 103 within HLA-B. This residue is present at the end of a b-sheet while in contact with a few hydrophobic residues from the helix above and may be important for maintaining the structural scaffold and creating the binding surface We created this figure using PDB ID (1K5N); **(C)** Atoms in residues 73 and 90 are shown as spheres. HLA-C (gray) and β-2-macroglobulin (wheat) are shown as ribbons. The small binding peptide is in direct contact with residue 73, and residue 90 is located in a loop region just after the helix present on the peptide-binding surface. We created this figure using PDB ID (6JT0).

The crystal structures in Figure 5 demonstrate that *HLA-DRB1* residues at 37 and 38 are located on the peptide-binding surface. Residue 103 within *HLA-B*, which appears to interact with a peptide, may play an important role in maintaining the structural scaffold and creating a binding surface. Within *HLA-C*, residues 73 and 90 are located on the peptide-binding groove.

## 4 Discussion

### 4.1 Findings

Previous epidemiological studies and GWAS have identified potential biological mechanisms underlying asthma, but few studies have focused on late-onset asthma. Prior GWAS have identified variants within the MHC region that are significantly associated with various asthma phenotypes (Li et al., 2010; Galanter et al., 2014). The MHC locus is the most gene-diverse and gene-dense region of the human genome, and the genetic architecture of the region is shaped by multiple factors, including gene shuffling and selective pressure in response to migration-related exposure to various environmental pathogens (Prugnolle et al., 2005; Traherne et al., 2006). We propose that racial and ethnic differences in allele frequencies in the MHC region of the genome may contribute, at least in part, to the observed racial disparities in asthma.

We leveraged a multi-ancestry cohort and WGS data to identify race/ethnicity-specific genetic variants associated with late-onset asthma. This is one of only a few studies that have conducted race/ethnicity-specific fine-mapping of the MHC region in the context of late-onset asthma in a United States-based cohort (Daya et al., 2021). We discovered several candidate gene variants associated with late-onset asthma that appear to play roles in allergy- and atopy-mediated airway inflammation and hyperresponsiveness. Additionally, we discovered race/ethnicity-specific candidate variants involved in immune pathways.

**FIGURE 6:**
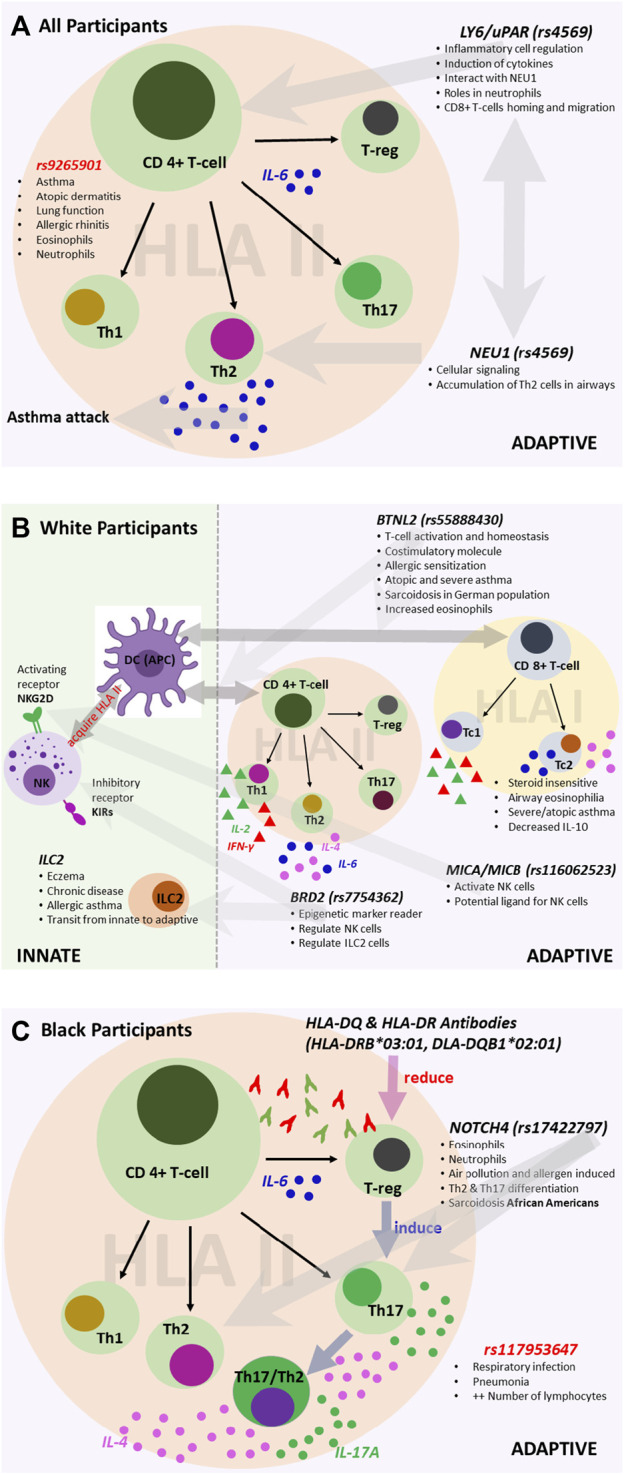
Variants associated with the immune system for **(A)** all participants, **(B)** White participants, and **(C)** Black participants. We reconstructed each pathway based on findings from the literature and sources that include the UK Biobank and Finnish Biobank. The figures illustrate the potential roles of the associated variants.

As additional validation of our findings, the lead variants we discovered are also well-established expressed quantitative trait loci (eQTL). For all participants, we discovered variants associated with both innate and adaptive immunity, primarily allergy- and atopy (eczema)-prone pathways (Figure 6). The 3′UTR *LY6G5C* variant *rs4569* is inversely associated with expression of *LY6G5C* and *LY6G5B*, which are members of the lymphocyte antigen superfamily *LY6/uPAR* (Loughner et al., 2016), both in lung tissue (*p* = 1.8 × 10^−43^; *p* = 1.2 × 10^−29^, respectively) and whole blood (*p* = 1.3 × 10^−119^; *p* = 1.8 × 10^−33^, respectively) (Supplementary Table S4). *LY6* protein has previously been characterized as playing a critical role in inflammatory cell regulation, including activation, proliferation, migration, interactions between cells, maturation of antigen-presenting cells (dendritic cells and macrophages), and induction of cytokines upon antigen challenges due to infection and various environmental stimuli (Lee et al., 2013; Loughner et al., 2016). Additionally, publicly available chromatin conformation data (Hi-C) suggest that *rs4569* is in a long-range distal region that interacts with the *NEU1* gene in mesenchymal stem cells (MES) (Supplementary Table S5). The enzymatic activity of *NEU1* increases the signaling of T helper 2 (T_H_2) cells and was found to infiltrate and accumulate in the airways and inflamed areas in murine models during acute asthma attacks and children with asthma after viral infection (Katoh et al., 2010; Pech et al., 2018).

For White participants, SNPs and HLA alleles identified from fine-mapping of the MHC region were also associated with genes that regulate innate and adaptive immune responses, particularly pathways involving atopy-related and allergen-driven airway inflammation and hyperresponsiveness (Figure 6). The lead SNP, *rs55888430*, has been shown to interact with the butyrophilin-like 2 (*BTNL2*) gene in IMR90 and MES cell lines (Supplementary Table S5) (Martin et al., 2017). *BTNL2* has also been associated with atopic asthma in children in Korea, dust-mite-specific IgE response in a Japanese population, and sarcoidosis in a White German population (Rybicki et al., 2005; Konno et al., 2009; Kim et al., 2021). The regulatory role of *BTNL2* was demonstrated in T cell activation and homeostasis as a costimulatory molecule during HLA class II antigen presentation to T cell receptors (Konno et al., 2009). Further, variants identified from the conditional analysis in White participants, namely, *rs116062523* and *rs7754362*, are eQTLs associated with the expression of *MICA/MICB* and *BRD2*, respectively. *MICA/MICB* and *BRD2* influence the activation and function of natural killer (NK) cells as a potential ligand and through the recognition of epigenetic markers, respectively (Stephens, 2001; Kerscher et al., 2019). Depending on the environment, NK cells can directly and indirectly regulate both activation and inhibition of CD4^+^ and CD8^+^ T cells (Vivier et al., 2008; Pallmer and Oxenius, 2016). Recent studies have shown that like CD4^+^ T helper 1 (T_H_1) and T_H_2 cells, some CD8^+^ subtypes, including cytotoxic type 1 and type 2 T cells (T_C_1 and T_C_2), can also produce type 1 and 2 cytokines (Lourenço et al., 2016). Some studies have shown that T_C_2 cells are more strongly associated with corticosteroid insensitivity, persistent airway eosinophilia, severe asthma, and atopic asthma than CD4^+^ T_H_2 cells (Gelfand and Hinks, 2019). For White participants, there was notable interaction and/or transition between innate and adaptive immunity involving NK cell-dependent CD4^+^ and CD8^+^ T cell responses. Similar variants (and/or interactions) have not been previously reported in studies of early-onset asthma in a United States-based cohort stratified by race/ethnicity.

For Black participants, the HLA-DRB1*03:01 and HLA-DQB1*02:01 alleles were significantly associated with late-onset asthma. Most HLA II-associated autoimmune diseases are accounted for by HLA-DR2DQ6, HLA-DR4DQ8, and HLA-DR3DQ2 haplotypes, and HLA II alleles are much more diverse or polymorphic in African populations compared to White European populations (Mangalam et al., 2013). The HLA analysis results suggest that for Black late-onset asthma cases, T_H_2 and T helper 17 (T_H_17) cell-mediated pathways may play a role (Figure 6) (Harb et al., 2021). When inflammation occurs due to the presence of an HLA antibody, cells expressing HLA-DQ and HLA-DR activate CD4^+^ T helper cells and promote the conversion of functional T regulatory cells (T_reg_) to T_H_17 T cells (Mangalam et al., 2013; Lion et al., 2016). Moreover, the plasticity of T_H_17 cells allows the conversion of human T_H_17 T cells into cells that produce both T_H_2 and T_H_17-related cytokines, including IL-4 and IL-17A, respectively (Cosmi et al., 2011). An intron variant, *rs17198965*, approximately 36,316 bp away from the canonical transcription start site of HLA-C, had a strong promoter-like signature in lung tissue, bronchial epithelial cells, CD8^+^ T cells, CD4^+^ and T_H_17 T cells, and B lymphocytes (cCRE accession ID: EH38E3700474). Further, *rs17198965* resides in the binding motif for the transcription factor *EZH2*, which suppresses major MHC class I molecule expression and is crucial for initiating CD4^+^ T cell-associated response upon viral infection (Karantanos et al., 2016).

It is well-established that T_H_2 and T_H_17 cells mediate airway inflammation. The results of our conditional analyses are consistent with this and further show that the selected variant rs17422797 is associated with increased numbers of both eosinophils and neutrophils (βeta = 0.04, *p* = 1.43 × 10^−20^; βeta = 0.03, *p* = 3.65 × 10^−18^, respectively) (Broad Institute, 2021). Mechanistically, exposure to allergens and particulate matter promotes T_H_2 and T_H_17 T cell differentiation in a manner dependent on the interaction of *NOTCH4* receptors on T_reg_ cells with the Notch receptor ligand, *Jagged 1,* on antigen-presenting cells (Xia et al., 2018; Harb et al., 2021). Previous work has found associations in African Americans carrying *NOTCH4*-associated variants with sarcoidosis, which involves the formation of inflammatory cells (granulomas) in the body, primarily in the lungs (Adrianto et al., 2012).

African American individuals with asthma have an increased burden of symptoms and increased morbidity and mortality compared to White individuals with asthma and display resistance to inhaled corticosteroid treatment (Barnes et al., 2019). T_H_17 is also linked to a difficult-to-treat, steroid-insensitive asthma phenotype (Chesné et al., 2014) and has been associated with difficult-to-control asthma in inner-city African American children and atopic dermatitis in African American individuals (Brown et al., 2017). Our findings propose candidate pathways that may be involved in the etiology of asthma in this population, related to population-specific allele frequencies and potential gene-gene and gene-environment interactions.

For HLA-DRB, HLA-B, and HLA-C, we examined the crystal structures with the highest resolution to determine the presence of functional consequences associated with selected individual residues in some alleles. Residues L37 and L/V38 of HLA-DRB are located in the peptide-binding surface and are in contact with the bound peptide. One of the two residues is also in contact with a helix from HLA-DRA that comprises the HLA complex. In HLA-B, L/V103 is an anchor residue of the helix that comprises the peptide-binding cavity and contributes to the stability of the hydrophobic patch composed of residues L109, V165, L168, and L172. Of the two residues selected from HLA-C, T73 is in direct contact with the peptide-binding surface while residue A90 is located in a loop region adjacent to the helix on the peptide-binding surface.

### 4.2 Strengths and limitations

The current study has several strengths. First, the collection of next-generation WGS data as opposed to genome-wide microarray-based ChIP-chip data enables access to comprehensive information at an allelic level across the diverse MHC locus. Second, enabled by the ancestry diversity in the PEGS cohort, we took advantage of natural differences in genomic LD across diverse populations to conduct transethnic gene mapping, enabling the prioritization of candidate genes, fine-mapping of functional variants, and potential identification of SNPs associated with disease risk in admixed populations.

Despite these strengths, the results must be interpreted in the context of important limitations. First, the importance of the sociocultural context of the varying results for Black and White subgroups cannot be understated. Race is a social construct correlated with health, economic, and exposure disparities. While socioeconomic status and other aspects of health disparities are important risk factors for many diseases, they are both a component and confounder of genetic associations and require further investigation (Fiscella and Williams, 2004). While genes and their variants are involved in the genetic etiology of asthma, regardless of race/ethnicity, the frequency of variants differs across strata, thus yielding varying results for significant associations. Additionally, the role of the environment, including structural racism, is crucially important, and gene-gene and gene-environment interactions may contribute to differing associations. Additionally, the small sample sizes of some of the stratified analyses may have affected statistical power, so the results should be interpreted with appropriate caution. Finally, we defined asthma cases using information on self-reported physician diagnoses. Given that asthma can be both over- and underdiagnosed (Aaron et al., 2018), this represents a potential confounder.

## 5 Conclusion

In this study, we explored the pathological role of genetic variants within the MHC region in late-onset asthma due to their critical importance in immune function/response and highly polymorphic nature. Our results suggest interesting parallels and contrasts in the genes and pathways that are associated with asthma dependent on race/ethnicity. The association of HLA II-associated genes and T_H_2-related genes with asthma regardless of race/ethnicity may support a basic “minimum requirement” immune component to asthma that comprises increased susceptibility to sensitization and T_H_2 activation. Various polymorphisms, depending on ancestry, may reflect stochastic genetic events (such as different allele frequencies) in populations but mechanistically induce similar effects. In contrast, in our cohort, we found a strong association between T_H_17 pathways and late-onset asthma in Black participants. Our findings have important clinical implications because knowledge of race/ethnicity-specific pathways for complex diseases such as late-onset asthma can help further dissect the etiology of the trait, which can ultimately assist with the development of precise, targeted treatments.

## Data Availability

Publicly available datasets were analyzed in this study. This data can be found here: https://gtexportal.org/home/ and https://www.ukbiobank.ac.uk/.
