# Supplementary material for: Race/ethnicity-stratified fine-mapping of the MHC locus reveals genetic variants associated with late-onset asthma
Source: Front Genet. 2023 Jun 21;14:1173676. doi: 10.3389/fgene.2023.1173676 (PMC10321602; doi:10.3389/fgene.2023.1173676)
Supplement: Supplementary file 1 [file DataSheet1.pdf]

Supplementary Material

Race/Ethnicity-Stratified Fine-Mapping of the MHC Locus Reveals Genetic Variants Associated with Late-Onset Asthma

Eunice Y. Lee, Wonson Choi, Adam B. Burkholder, Lalith Perera, Jasmine A. Mack, Frederick W. Miller, Michael B. Fessler, Donald N. Cook, Peer W. F. Karmaus, Hideki Nakano, Stavros Garantziotis, Jennifer H. Madenspacher, John, S. House, Farida S. Akhtari, Charles S. Schmitt, David C. Fargo, Janet E. Hall, Alison A. Motsinger-Reif[1*]

*** Correspondence:** Alison A. Motsinger-Reif: alison.motsinger-reif@nih.gov

1 Supplementary data

1.1 Study participants: PEGS

The Personalized Environmental and Genetics Study (PEGS) (N=19,672) collects questionnaire-based data on both endogenous and exogenous exposures to investigate the relationships between the exposome, genomics, and human health. Beginning in 2002, study participants were recruited through healthcare clinics, university campuses, health fairs, and volunteer study drives at businesses, community centers, and events in North Carolina. At enrollment, participants provide details of their medical, surgical, and health history, including current and past diseases and medical conditions. A description of the PEGS study design and the questionnaires administered to participants can be found at https://joinastudy.niehs.nih.gov/studies/pegs.

1.2 Whole-genome sequencing data generation, processing, and quality control

Whole-genome sequencing (WGS) was performed by the Broad Institute on the NovaSeq 6000 platform to a minimum of 30X mean genome coverage. Details on whole-genome sequencing, variant calling, library construction, read processing, and sequence data quality control are described elsewhere (Akhtari et al., 2023). GATK 3.5.0 HaplotypeCaller, GATK 4.1.4.0 GenotypeGvcfs, and GATK 4.1.1.0 VariantFiltration were used to obtain variant calls, joint genotypes, and filtered flags, respectively. Variant calls were aligned to the GRCh38 reference genome using BWA-MEM (Li and Durbin, 2010). Variants with a heterozygosity phred score of <= 54.69 and VSQLOD score greater than 99.0% of true positive indels and 99.7% of true single nucleotide polymorphisms (SNPs) were assigned a 'PASS'. In this study, we excluded indels and focused on biallelic SNPs. We used PLINK 2.0 (Purcell et al., 2007) to conduct data quality control for the variants falling within the boundary of Chr6:28,510,120-33,480,577. We filtered out SNPs with a call rate less than 95%, minor allele frequency less than 1%, and those that failed the Hardy-Weinberg test at a 0.0001 significance-level threshold. For quality control of study participants, we excluded individuals with genotypes with more than 5% missing and pairs of individuals who were genetically related to the equivalent of second cousin (PLINK flags --genome --min 0.025). After quality control of WGS data, 40,535 SNPs remained.

1.3 Local genetic ancestry estimates

We used SHAPEIT 4.2.1 to perform haplotype phasing across 29,062,806 variants for the subgroup of PEGS participants with whole-genome sequencing data (n = 4,737), considering only variants that were genotyped in both the reference panel and PEGS. We estimated genome-wide local ancestry on the autosomes and X chromosome using RFMix, a discriminative modeling approach (Maples et al., 2013). The reference population was 2,504 unrelated individuals from the 1000 Genomes Project, Phase 3, with sequencing to a targeted depth of 30X (Byrska-Bishop et al., 2022). Based on the 1000 Genomes data, we classified genomic positions by five continental super populations: AFR (African), AMR (Admixed American), EAS (East Asian), EUR (European), and SAS (South Asian). Granular population groupings can be found in the International Genome Sample Resource (IGSR) (Fairley et al., 2020). We classified individuals as one of the five continental ancestral groups if any genome-wide local ancestral proportion was greater than 0.50.

1.4 MHC region-wide allelic association tests

After quality control of sequencing data for the major histocompatibility complex (MHC) region, 41,012, 39,867, and 47,433 SNPs remained for pooled, EUR, and AFR ancestry, respectively. To correct for multiple hypothesis testing, we adopted a similar approach as previous studies (Lee et al., 2020). We used the *coda* package in R to determine the effective number of tests by fitting an autoregression model to the summary statistics. The effective number of tests is often smaller than the empirical number of tests since it accounts for the local genetic correlation between SNPs (linkage disequilibHCG17 rium) used in association tests.

1.5 Fine-mapping: conditional analysis

Using WGS data for PEGS participants, we conducted fine-mapping in the MHC region at 6p22.1-21.3 (chr6:28,510,120-33,480,577, assembly of GRCh38.p14) to identify candidate causal variants for late-onset asthma. From the results of the allelic association tests, we rank-ordered SNPs by their strength of association (p-values). We performed stepwise regression by adding one SNP at a time until the effect of the lead signal (i.e., the SNP with the smallest p-value from the allelic association tests) reached p >0.05. During regression modeling, we considered the local linkage disequilibrium structure by filtering out SNPs based on their pairwise correlation (r^2 >0.8). The final model included independent SNPs associated with late-onset asthma adjusted for other SNPs and covariates.

1.6 Functional annotation of genetic variants

For annotation, we used public data from ENCODE (ENCODE Project Consortium, 2012; Luo et al., 2020), HUGin (Martin et al., 2017), GTEx (Genotype-Tissue Expression (GTEx) Project, 2022), RegulomeDB (Boyle et al., 2012), Open Target Genetics (Ghoussaini et al., 2021; Mountjoy et al., 2021) to delineate the potential biological links between genetic variants and late-onset asthma in PEGS participants. We used quantitative measures of functional importance (CADD score and RegDB rank and score), DNase I hypersensitive site sequencing, histone modification ChIP-seq (H3K4me3, H3K27ac), transcription factor binding ChIP-seq (CTCF), long-range chromatin interactions (Hi-C), and expression quantitative trait loci (eQTL) data to investigate each SNP's potential genomic regulatory/functional role. Based on functional annotation, we prioritized SNPs with higher scores and supportive evidence as candidate causal variants contributing to late-onset asthma association signals.

1.7 Assembly of HLA alleles: Kourami

Kourami is a recently developed enrichment-free computational method that uses WGS data to directly assemble full sequences of the peptide-binding domain (exons 2 and 3 for class I human leukocyte antigen (HLA) genes and exon 2 for class II HLA genes) (Lee and Kingsford, 2018). We first built a reference panel using multiple sequence alignments of known HLA alleles from the Immuno Polymorphism Database-International ImMunoGeneTics (IPD-IMGT)/HLA project database (Robinson et al., 2020). Based on the reference panel, we constructed an initial directed acyclic HLA graph, which we further modified by alignment projection (deletion or mismatch, insertion into a gap column, or insertion into a new column). Using this modified HLA graph, we identified the best paths with the maximum weights (number of reads) and most supportive phasing information to assemble pairs of candidate HLA alleles. We filtered the assembled HLA alleles to exclude ambiguous calls where two or more haplotypes were considered equally likely as well as calls supported by a "MaxFlow" parameter less than 10 and those with less than 95% identity with the called haplotype sequence.

1.8 Amino acid position inference: CookHLA and HATK

The CookHLA and HLA analysis toolkit (HATK) enrichment-free computational HLA imputation/inference methods are data-based matching techniques that align WGS reads to known alleles. We used 1000 Genomes Project reference panels (SNPs in the MHC region and HLA types). For pooled ancestry (i.e., all study participants) (n=2,504), we used the ALL reference panel. For European and African ancestries, we used the EUR and AFR reference panels, respectively. We first inferred four-digit classical HLA alleles (*HLA-A*, *-B*, *-C*, *-DQB*, and *-DRB1*) with CookHLA. CookHLA uses the latest hidden Markov model, which incorporates genetic distance as an input. CookHLA also captures local genetic information through repeated imputation for each exon in HLA genes by embedding a marker set in the middle position of an exon and adaptively learning the genetic map of the MHC region. In this way, CookHLA accounts for variability in highly polymorphic exons and data- or population-specific linkage disequilibrium (LD) structure in the MHC region. The best-matched HLA alleles are generated as binary markers (presence/absence) based on consensus posterior probabilities from the repeated imputation.

We then applied HATK using the HLA allele information generated from CookHLA (the HLA PED equivalent to a plink PED file) to infer residues at each amino acid position. We built a dictionary for amino acid and DNA sequences for HLA genes based on the IMGT/HLA database and cleaned the HLA allele names according to the nomenclature defined by the World Health Organization
amino acid positions as binary markers (presence/absence) and then conducted logistic regression using this dosage data, adjusting for the same covariates as for pooled, EUR, and AFR ancestries.

2 Supplementary Figures and Tables

2.1 Supplementary Figures

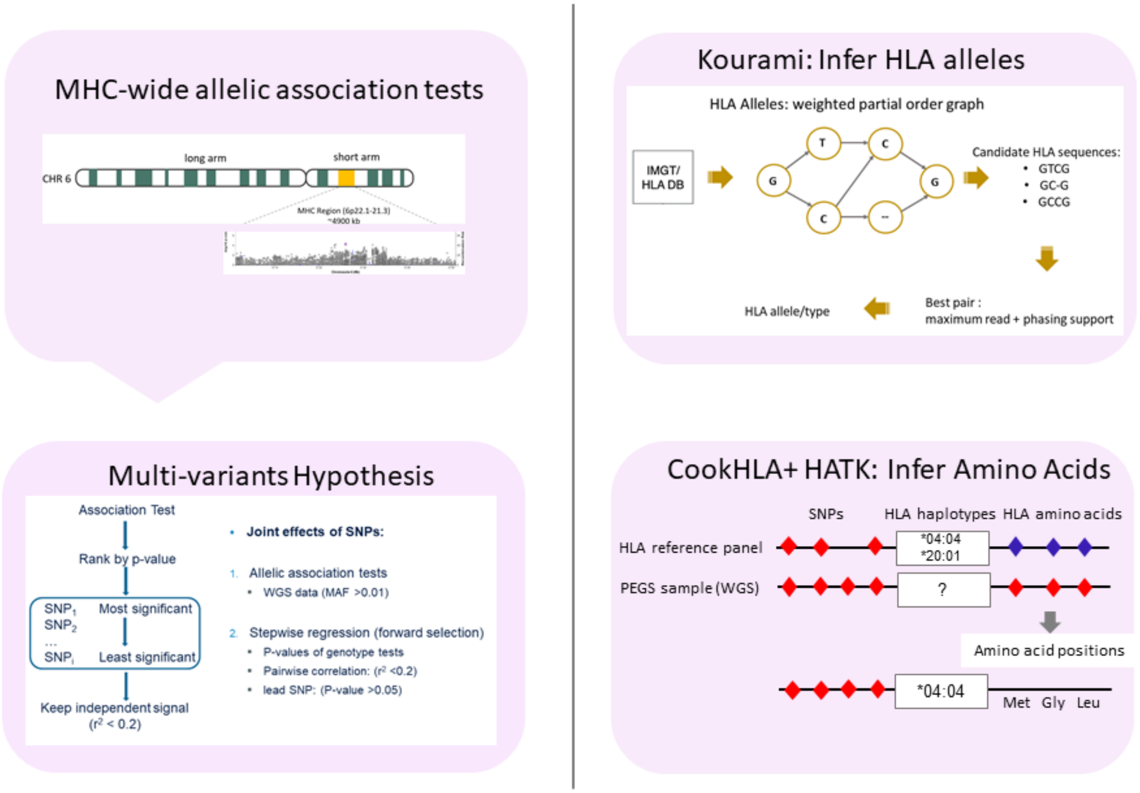

Supplementary Figure S1. Overview of the study analyses/methodologies.

(a)

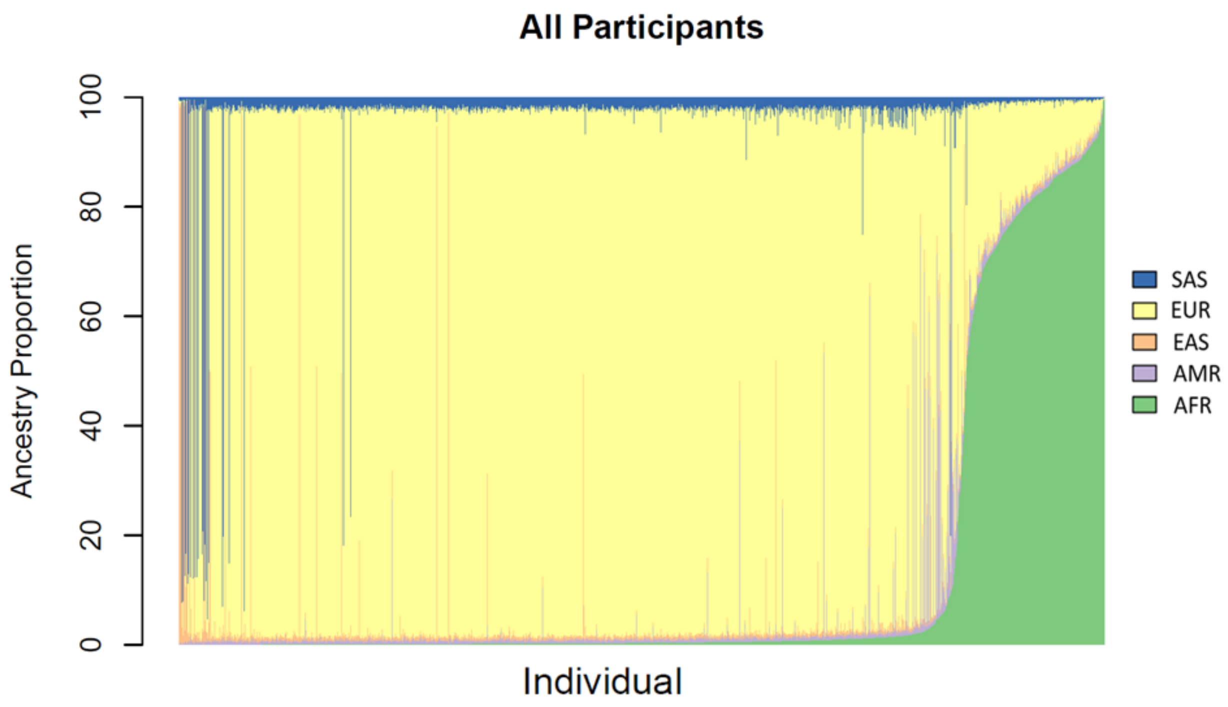

All Participants
Ancestry Proportion
0
20
40
60
80
100
Individual
SAS
EUR
EAS
AMR
AFR

(b)

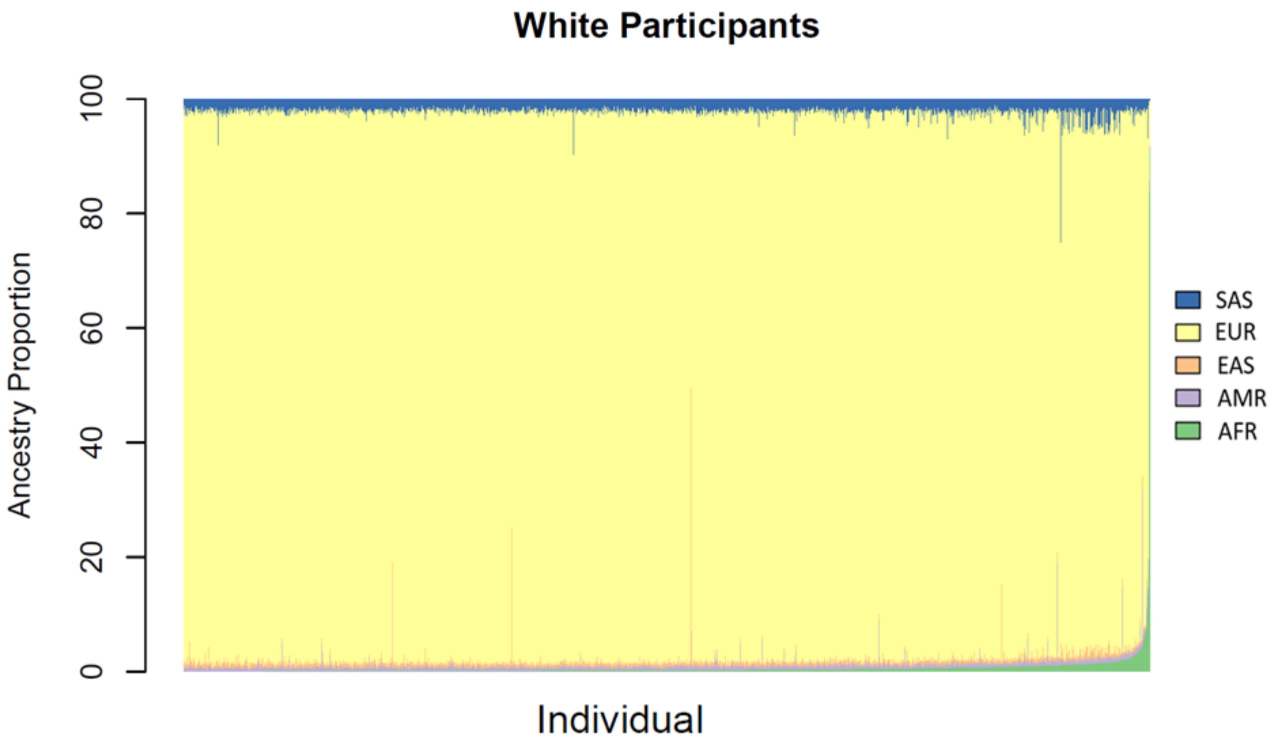

White Participants
Ancestry Proportion
0
20
40
60
80
100
Individual
SAS
EUR
EAS
AMR
AFR

(c)

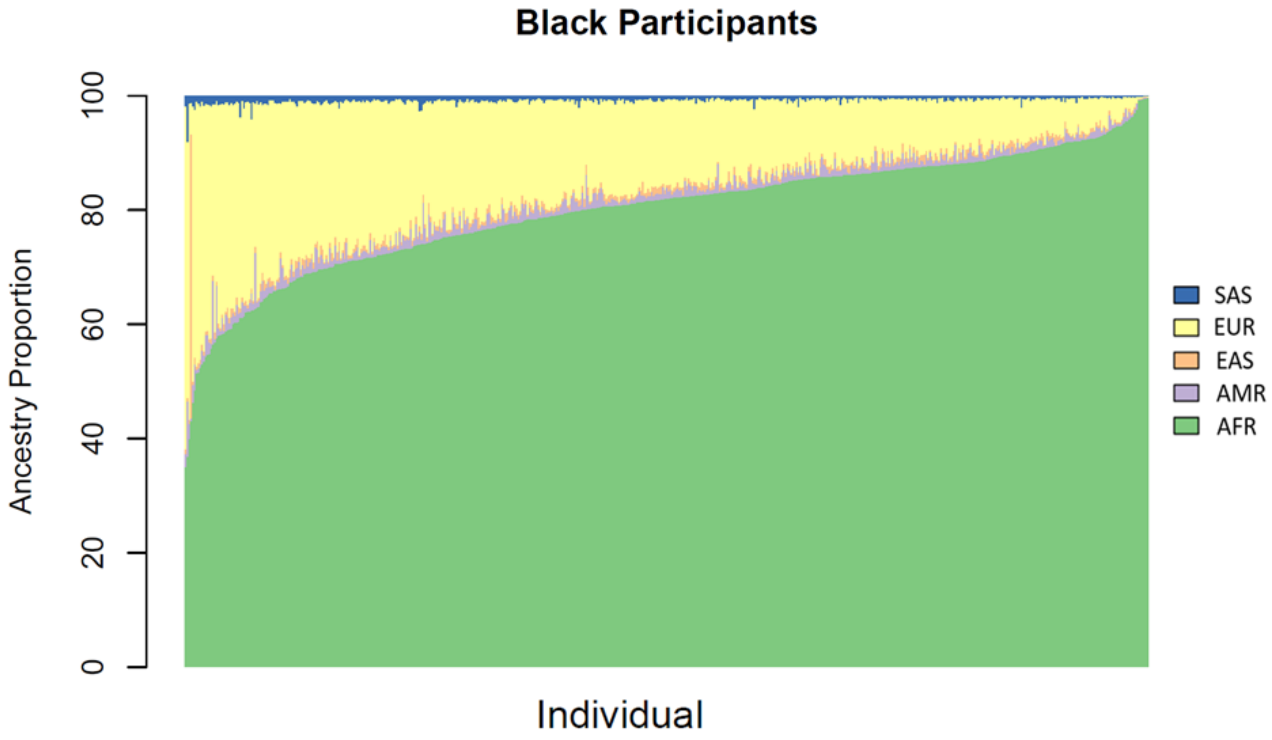
Black Participants
Ancestry Proportion
0
20
40
60
80
100
Individual
SAS
EUR
EAS
AMR
AFR

(d)

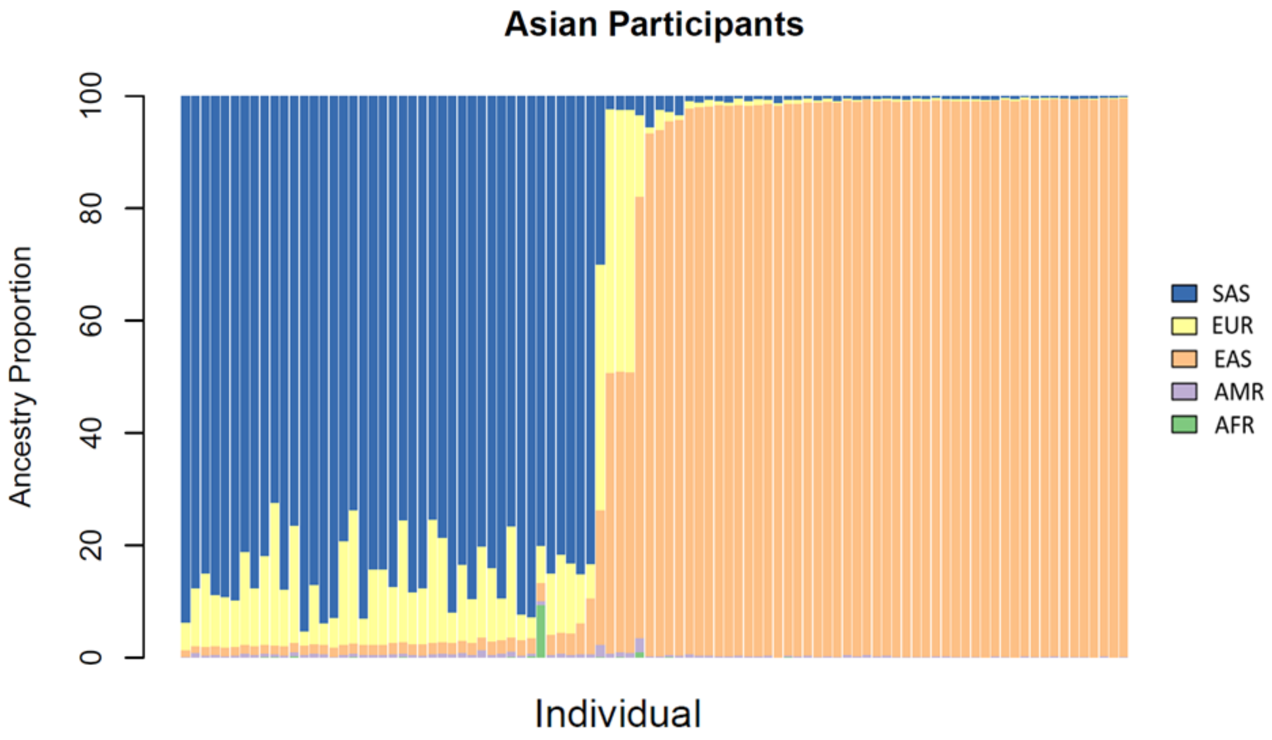
Asian Participants
Ancestry Proportion
0
20
40
60
80
100
Individual
SAS
EUR
EAS
AMR
AFR

(e)

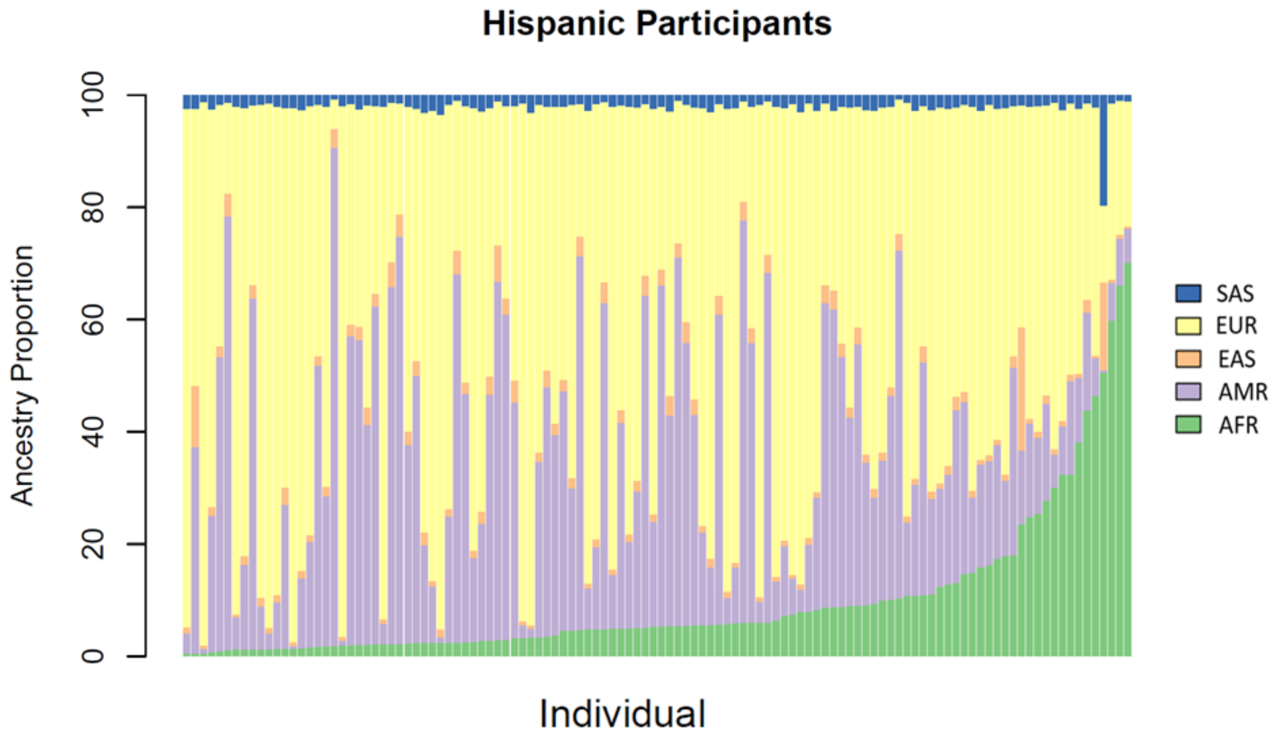
Hispanic Participants
Ancestry Proportion
0
20
40
60
80
100
Individual
SAS
EUR
EAS
AMR
AFR

(f)

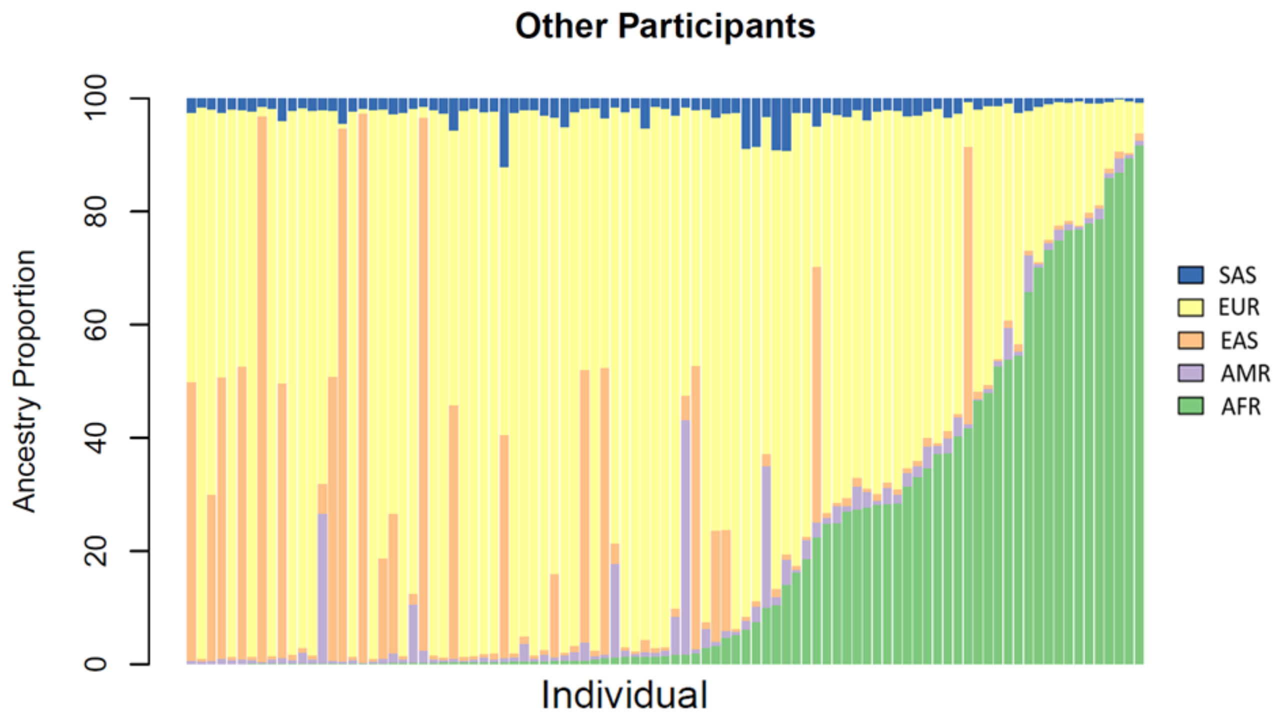
Other Participants
Ancestry Proportion
0
20
40
60
80
100
Individual
SAS
EUR
EAS
AMR
AFR

Supplementary Figure S2. Genome-wide ancestry proportions for five super populations for PEGS participants: African (AFR), Admixed American (AMR), East Asian (EAS), European (EUR), and Southeast Asian (SAS). Each bar on the X-axis represents an individual participant. The Y-axis shows the variability of the five ancestry proportions for a given individual. The figures show the ancestry proportions for (a) all participants, (b) participants self-identifying as White, (c) participants self-identifying as Black, (d) participants self-identifying as Asian, (e) participants self-identifying as Hispanic, and (f) participants self-identifying as other races/ethnicities.

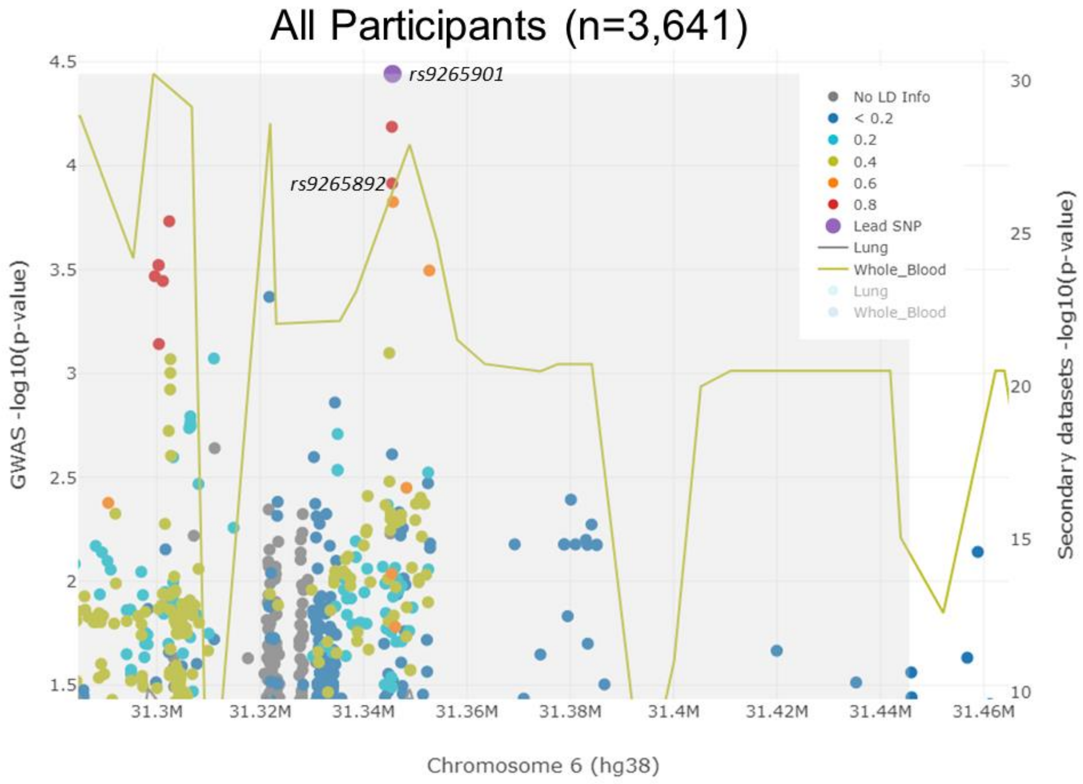
All Participants (n=3,641)
rs9265901
rs9265892
No LD Info
< 0.2
0.2
0.4
0.6
0.8
Lead SNP
Lung
Whole_Blood
Lung
Whole_Blood
GWAS -log10(p-value)
Secondary datasets -log10(p-value)
4.5
4
3.5
3
2.5
2
1.5
30
25
20
15
10
31.3M
31.32M
31.34M
31.36M
31.38M
31.4M
31.42M
31.44M
31.46M
Chromosome 6 (hg38)

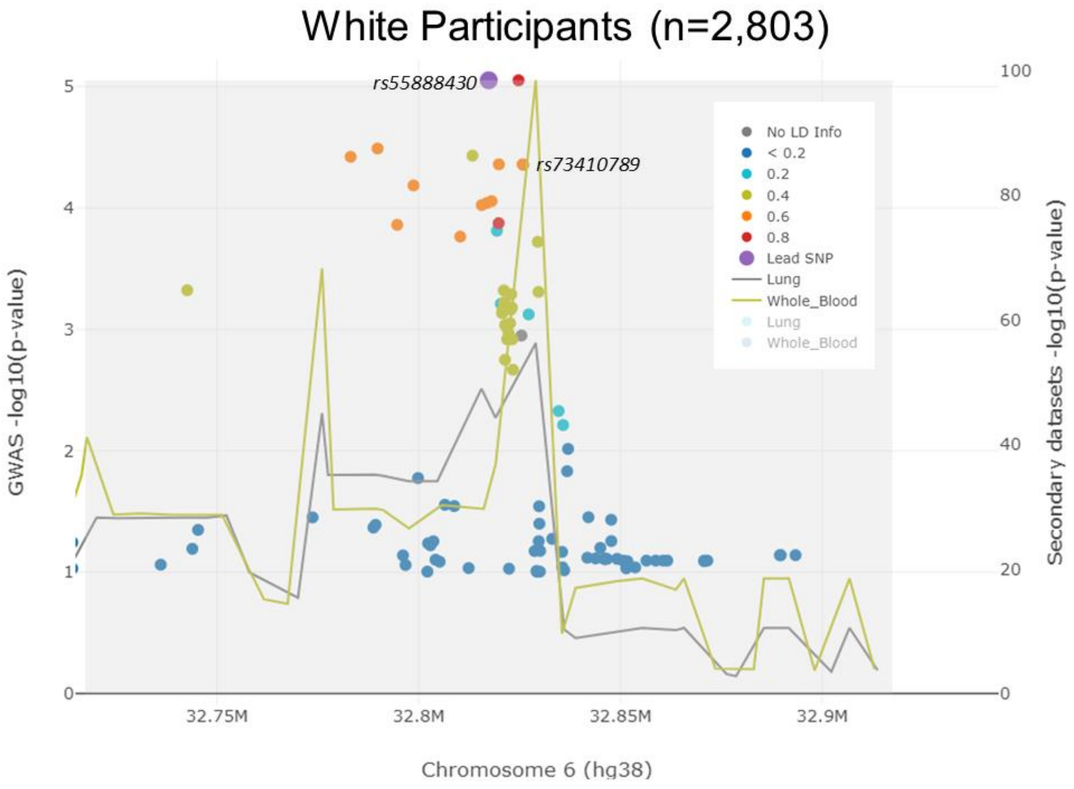
White Participants (n=2,803)
rs55888430
rs73410789
No LD Info
< 0.2
0.2
0.4
0.6
0.8
Lead SNP
Lung
Whole_Blood
Lung
Whole_Blood
GWAS -log10(p-value)
Secondary datasets -log10(p-value)
5
4
3
2
1
0
100
80
60
40
20
0
32.75M
32.8M
32.85M
32.9M
Chromosome 6 (hg38)

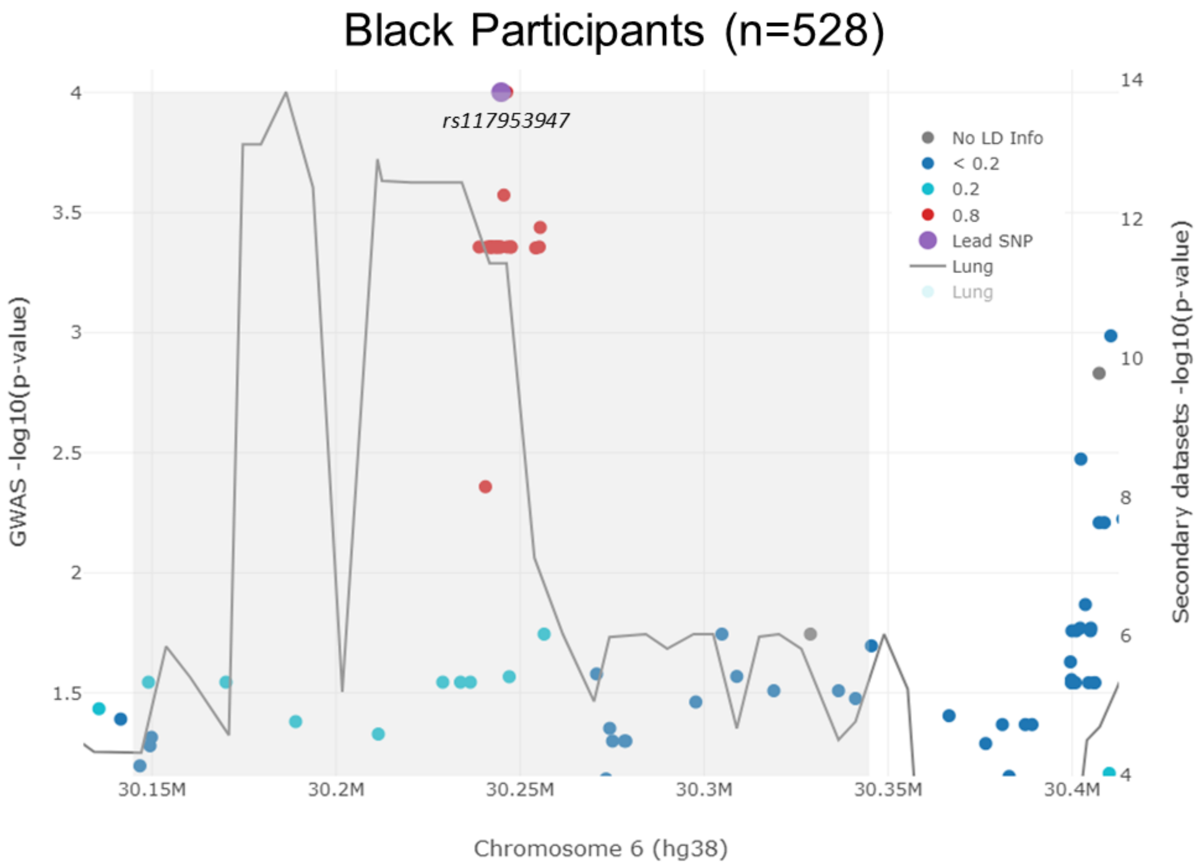

Supplementary Figure S3. LocusFocus plots of colocalization analysis results. The lead SNP for all participants (*rs9265901*) was not associated with the expression of *HLA-B* in lung tissue or whole blood. However, an SNP that was highly correlated with the lead *SNP (rs9265892*) was associated with the expression of *HLA-B* in whole blood. The lead SNP (*rs55888430)* for White participants was not associated with the expression of *HLA-DOB*. An LD SNP for the lead SNP (*rs73410789*) was associated with *HLA-DOB* expression in whole blood. The lead SNP for Black participants, *rs117953947*, was not associated with the expression of *HCG17* in lung tissue.

(a)

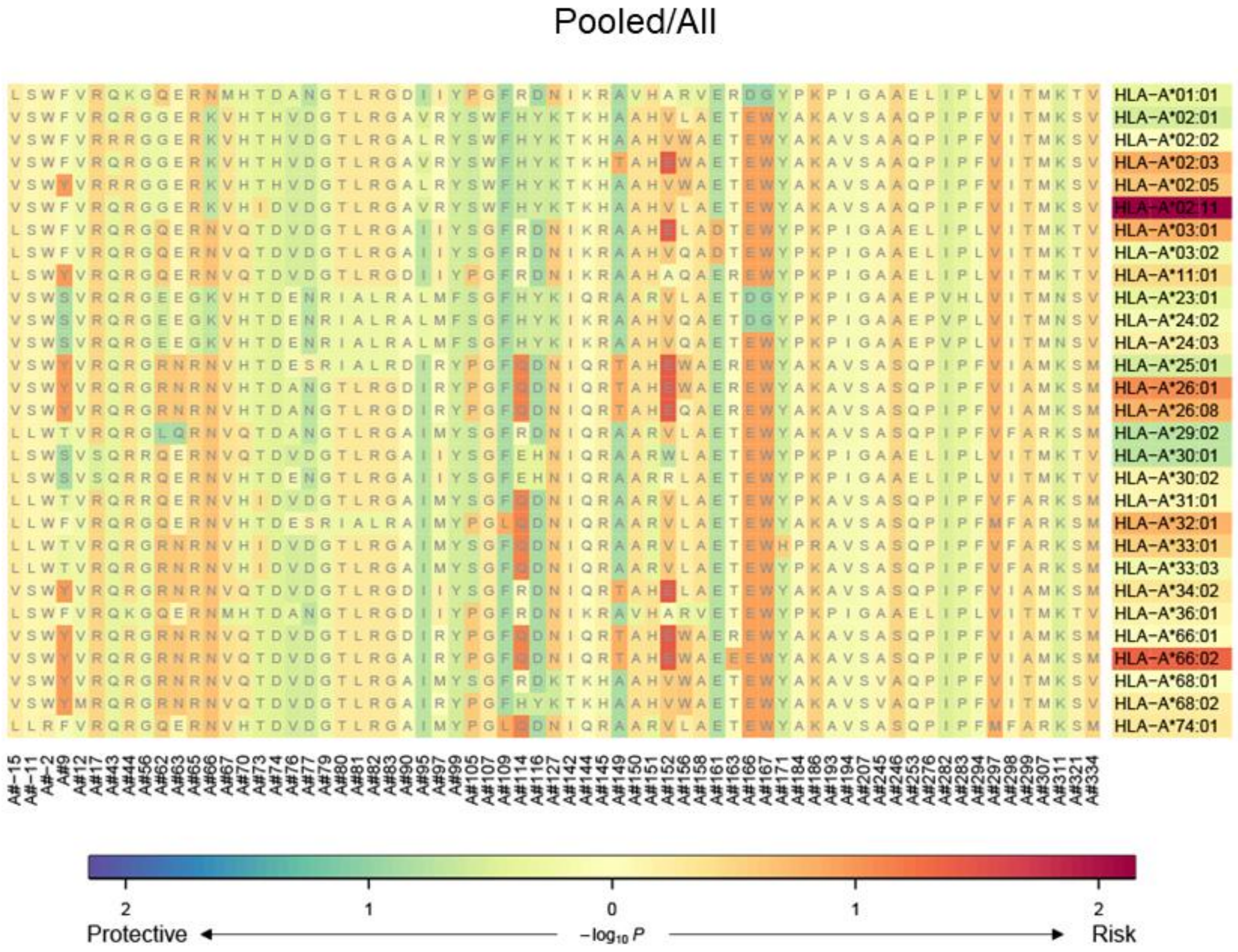
Pooled/All
HLA−A*01:01
HLA−A*02:01
HLA−A*02:02
HLA−A*02:03
HLA−A*02:05
HLA−A*02:11
HLA−A*03:01
HLA−A*03:02
HLA−A*11:01
HLA−A*23:01
HLA−A*24:02
HLA−A*24:03
HLA−A*25:01
HLA−A*26:01
HLA−A*26:08
HLA−A*29:02
HLA−A*30:01
HLA−A*30:02
HLA−A*31:01
HLA−A*32:01
HLA−A*33:01
HLA−A*33:03
HLA−A*34:02
HLA−A*36:01
HLA−A*66:01
HLA−A*66:02
HLA−A*68:01
HLA−A*68:02
HLA−A*74:01
2
1
0
1
2
Protective
−log10 P
Risk

(b)

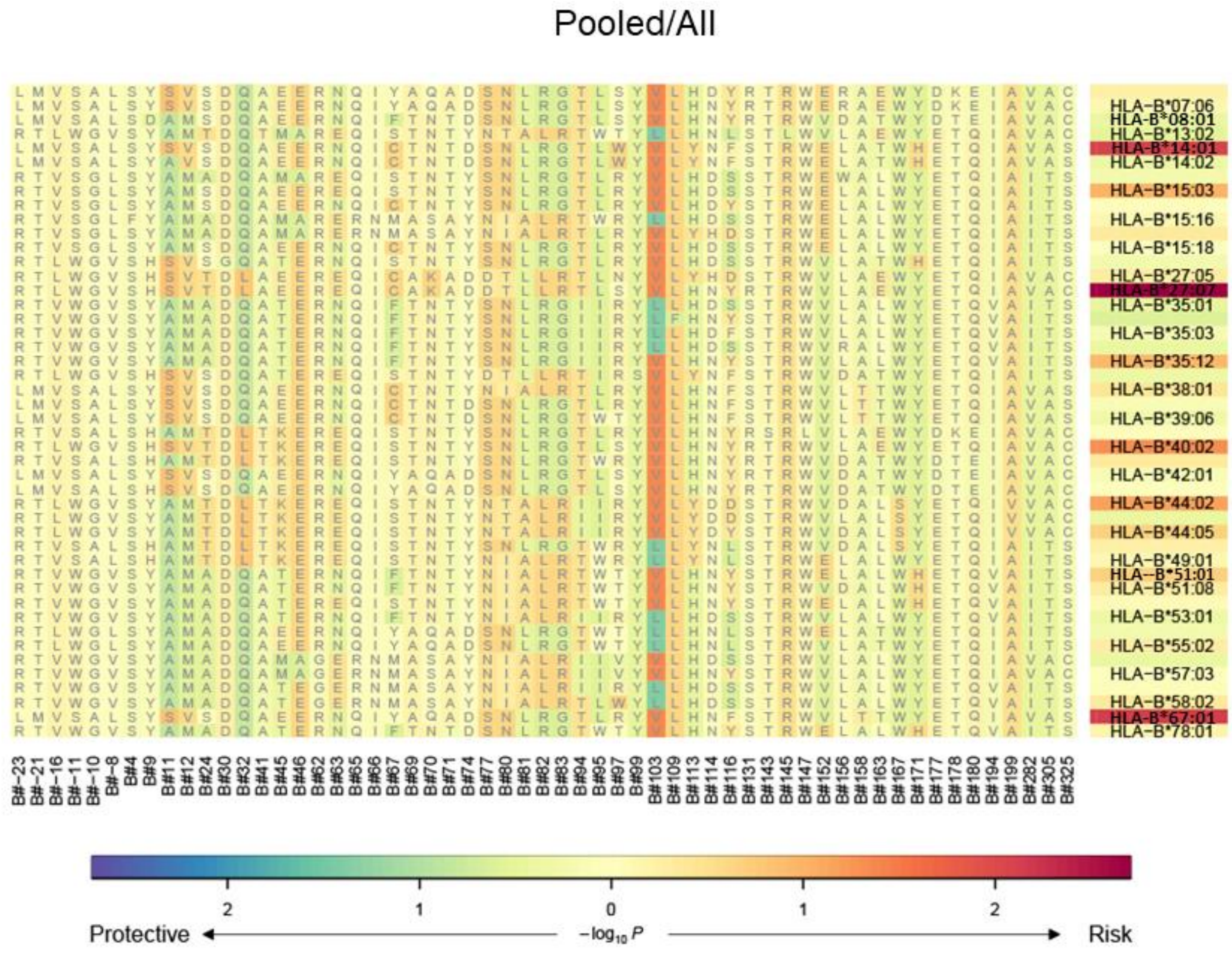
Pooled/All
HLA-B*07:06
HLA-B*08:01
HLA-B*13:02
HLA-B*14:01
HLA-B*14:02
HLA-B*15:03
HLA-B*15:16
HLA-B*15:18
HLA-B*27:05
HLA-B*27:07
HLA-B*35:01
HLA-B*35:03
HLA-B*35:12
HLA-B*38:01
HLA-B*39:06
HLA-B*40:02
HLA-B*42:01
HLA-B*44:02
HLA-B*44:05
HLA-B*49:01
HLA-B*51:01
HLA-B*51:08
HLA-B*53:01
HLA-B*55:02
HLA-B*57:03
HLA-B*58:02
HLA-B*67:01
HLA-B*78:01
2
1
0
1
2
Protective
−log10 P
Risk

(c)

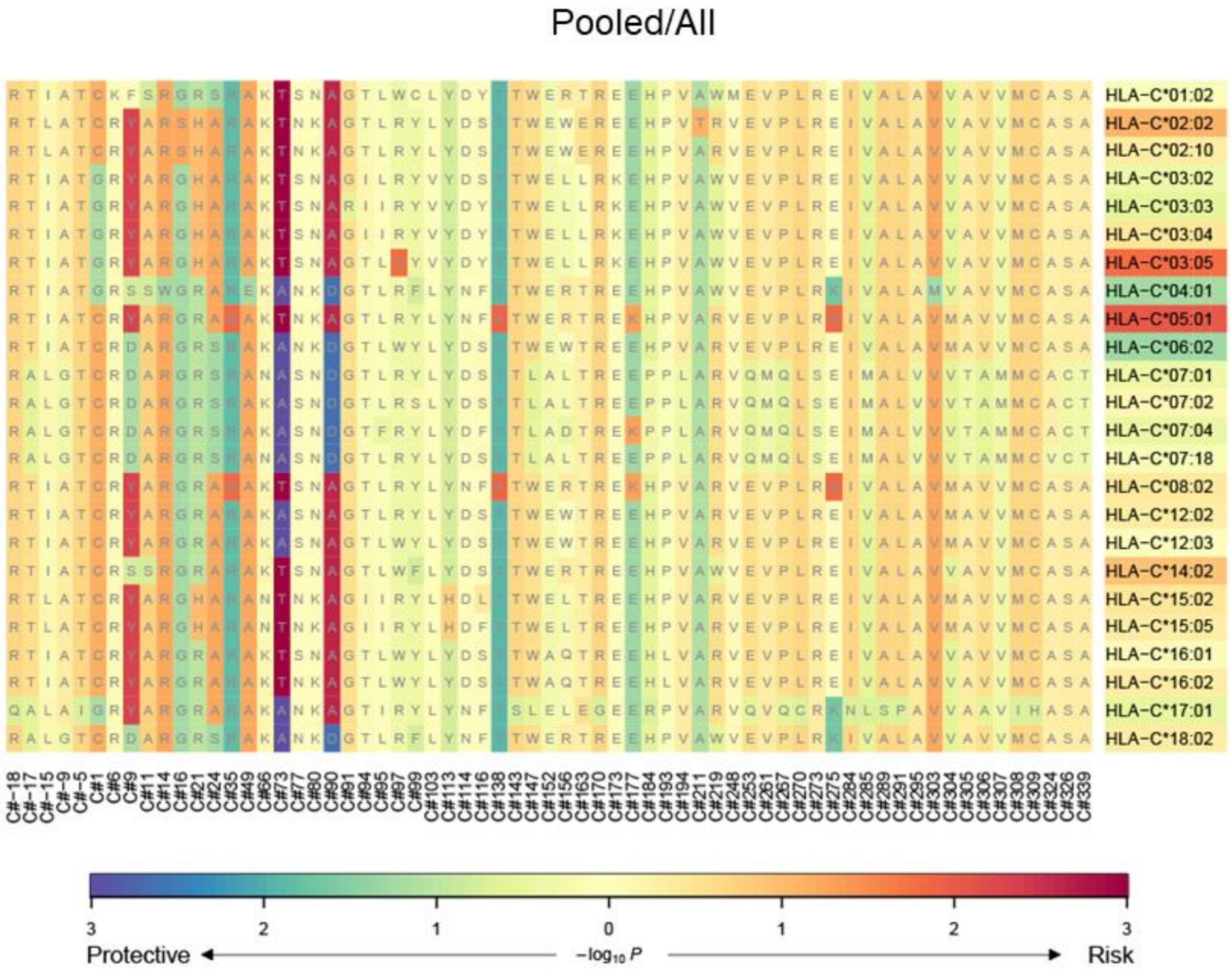

Pooled/All
HLA-C*01:02
HLA-C*02:02
HLA-C*02:10
HLA-C*03:02
HLA-C*03:03
HLA-C*03:04
HLA-C*03:05
HLA-C*04:01
HLA-C*05:01
HLA-C*06:02
HLA-C*07:01
HLA-C*07:02
HLA-C*07:04
HLA-C*07:18
HLA-C*08:02
HLA-C*12:02
HLA-C*12:03
HLA-C*14:02
HLA-C*15:02
HLA-C*15:05
HLA-C*16:01
HLA-C*16:02
HLA-C*17:01
HLA-C*18:02
3 2 1 0 1 2 3
Protective
−log10 P
Risk

(d)

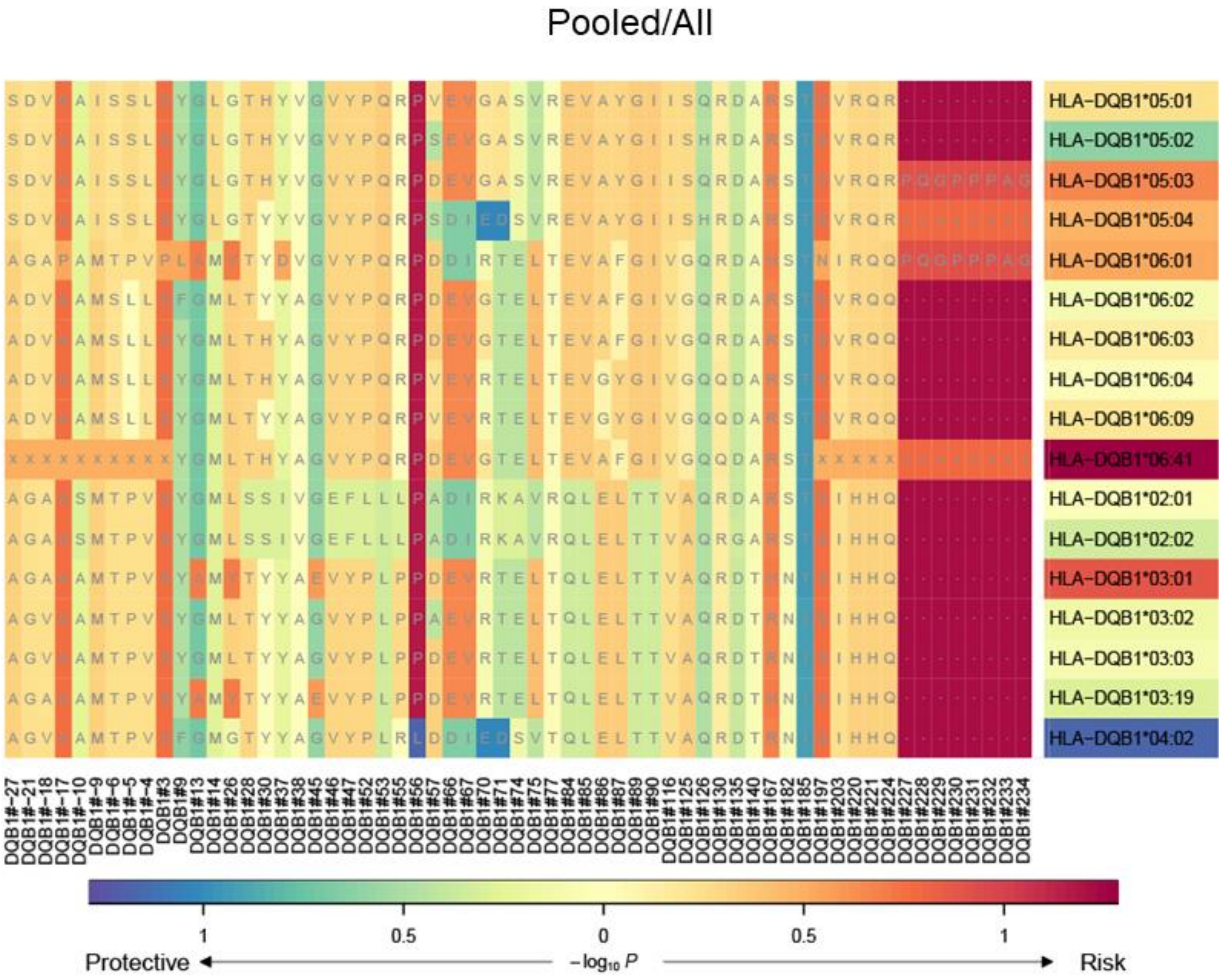
Pooled/All
HLA-DQB1*05:01
HLA-DQB1*05:02
HLA-DQB1*05:03
HLA-DQB1*05:04
HLA-DQB1*06:01
HLA-DQB1*06:02
HLA-DQB1*06:03
HLA-DQB1*06:04
HLA-DQB1*06:09
HLA-DQB1*06:41
HLA-DQB1*02:01
HLA-DQB1*02:02
HLA-DQB1*03:01
HLA-DQB1*03:02
HLA-DQB1*03:03
HLA-DQB1*03:19
HLA-DQB1*04:02
DQB1#-27
DQB1#-21
DQB1#-18
DQB1#-17
DQB1#-10
DQB1#-9
DQB1#-6
DQB1#-5
DQB1#-4
DQB1#3
DQB1#9
DQB1#13
DQB1#14
DQB1#26
DQB1#28
DQB1#30
DQB1#37
DQB1#38
DQB1#45
DQB1#46
DQB1#47
DQB1#52
DQB1#53
DQB1#55
DQB1#56
DQB1#57
DQB1#66
DQB1#67
DQB1#70
DQB1#71
DQB1#74
DQB1#75
DQB1#77
DQB1#84
DQB1#85
DQB1#86
DQB1#87
DQB1#89
DQB1#90
DQB1#116
DQB1#125
DQB1#126
DQB1#130
DQB1#135
DQB1#140
DQB1#167
DQB1#182
DQB1#185
DQB1#197
DQB1#203
DQB1#220
DQB1#221
DQB1#224
DQB1#227
DQB1#228
DQB1#229
DQB1#230
DQB1#231
DQB1#232
DQB1#233
DQB1#234
1
0.5
0
0.5
1
Protective
$-\log_{10} P$
Risk

(e)

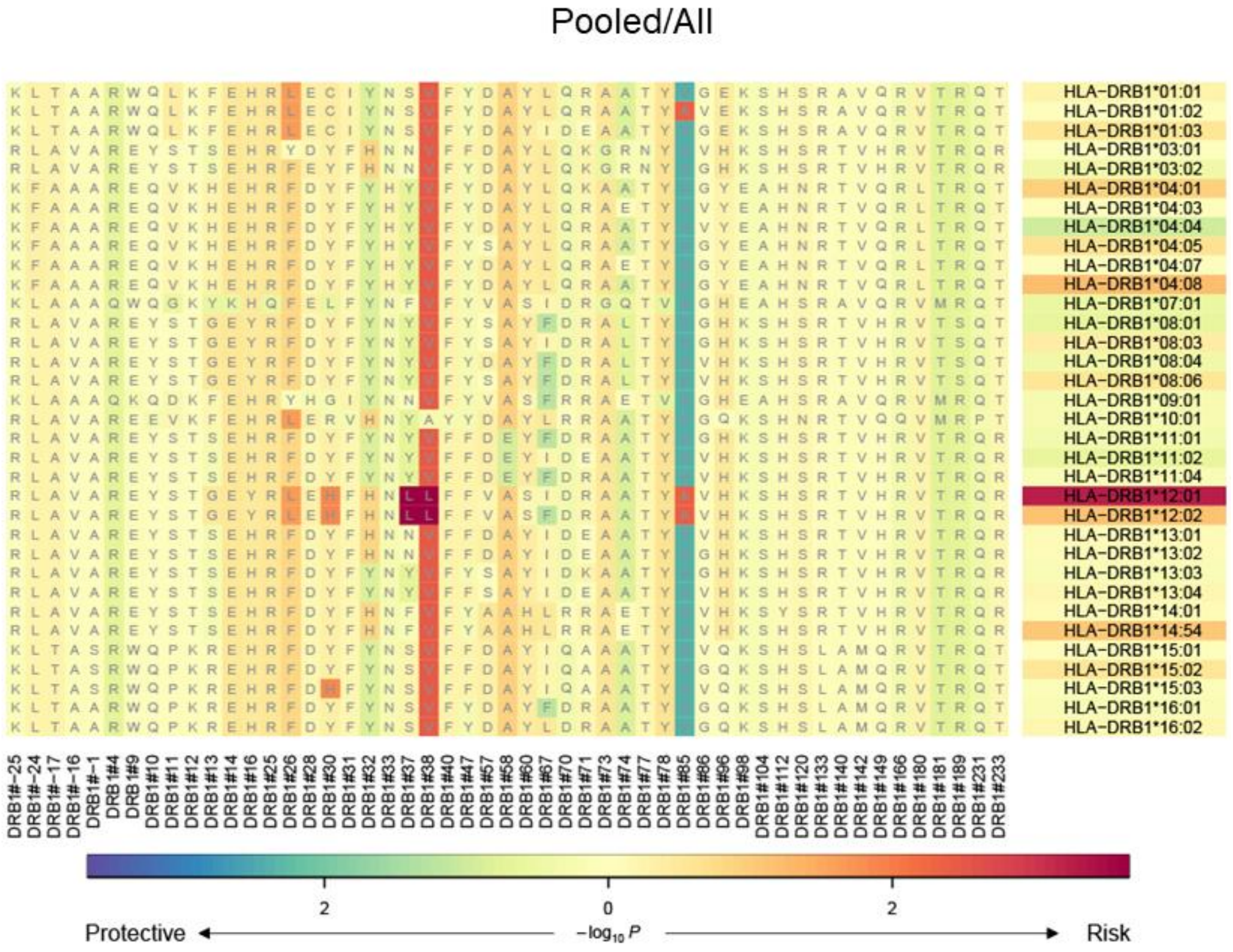

Pooled/All
HLA−DRB1*01:01
HLA−DRB1*01:02
HLA−DRB1*01:03
HLA−DRB1*03:01
HLA−DRB1*03:02
HLA−DRB1*04:01
HLA−DRB1*04:03
HLA−DRB1*04:04
HLA−DRB1*04:05
HLA−DRB1*04:07
HLA−DRB1*04:08
HLA−DRB1*07:01
HLA−DRB1*08:01
HLA−DRB1*08:03
HLA−DRB1*08:04
HLA−DRB1*08:06
HLA−DRB1*09:01
HLA−DRB1*10:01
HLA−DRB1*11:01
HLA−DRB1*11:02
HLA−DRB1*11:04
HLA−DRB1*12:01
HLA−DRB1*12:02
HLA−DRB1*13:01
HLA−DRB1*13:02
HLA−DRB1*13:03
HLA−DRB1*13:04
HLA−DRB1*14:01
HLA−DRB1*14:54
HLA−DRB1*15:01
HLA−DRB1*15:02
HLA−DRB1*15:03
HLA−DRB1*16:01
HLA−DRB1*16:02
DRB1#−25
DRB1#−24
DRB1#−17
DRB1#−16
DRB1#−1
DRB1#4
DRB1#9
DRB1#10
DRB1#11
DRB1#12
DRB1#13
DRB1#14
DRB1#16
DRB1#25
DRB1#26
DRB1#28
DRB1#30
DRB1#31
DRB1#32
DRB1#33
DRB1#37
DRB1#38
DRB1#40
DRB1#47
DRB1#57
DRB1#58
DRB1#60
DRB1#67
DRB1#70
DRB1#71
DRB1#73
DRB1#74
DRB1#77
DRB1#78
DRB1#85
DRB1#86
DRB1#96
DRB1#98
DRB1#104
DRB1#112
DRB1#120
DRB1#133
DRB1#140
DRB1#142
DRB1#149
DRB1#166
DRB1#180
DRB1#181
DRB1#189
DRB1#231
DRB1#233
2
0
2
Protective
$-\log_{10} P$
Risk

(f)

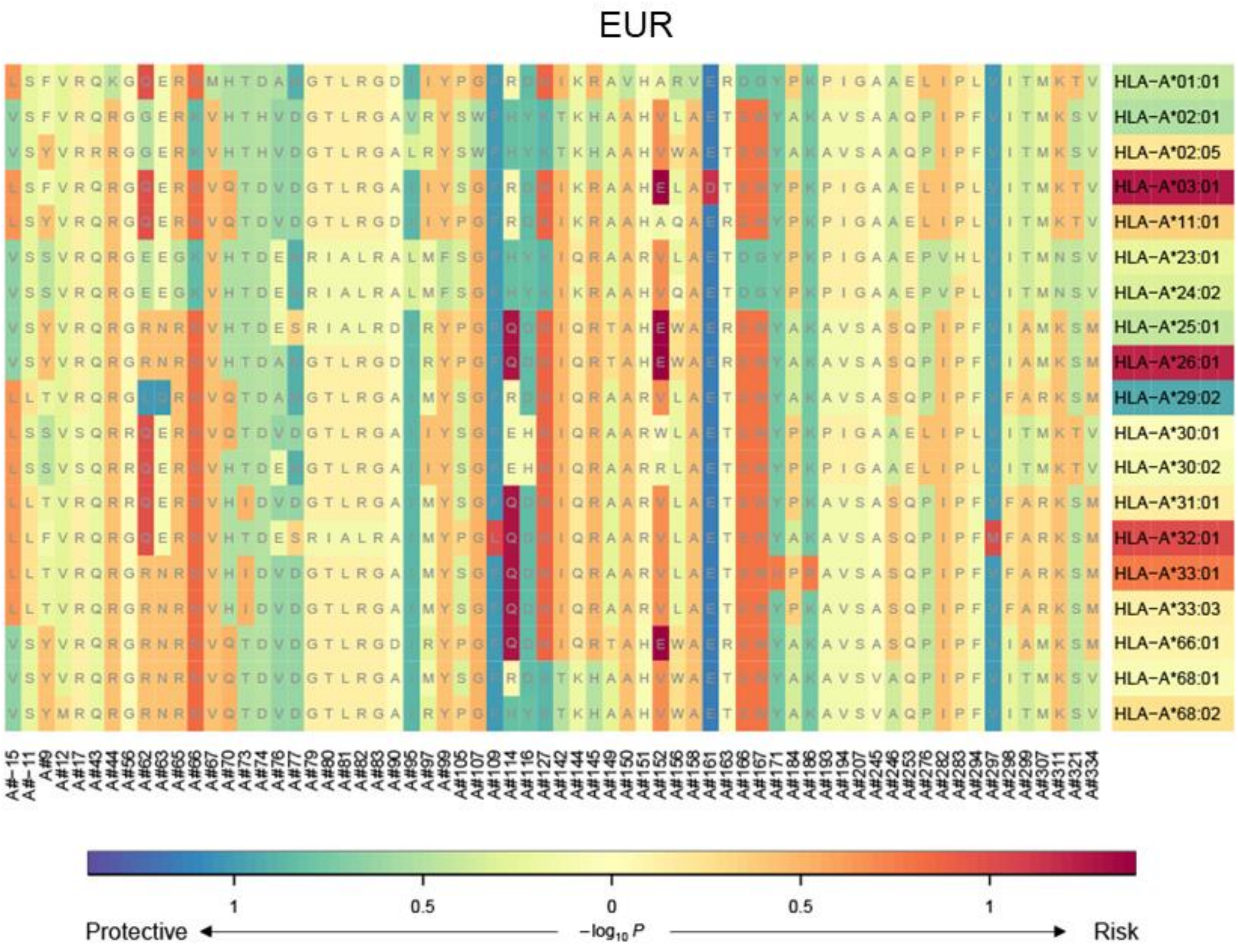
EUR
HLA-A*01:01
HLA-A*02:01
HLA-A*02:05
HLA-A*03:01
HLA-A*11:01
HLA-A*23:01
HLA-A*24:02
HLA-A*25:01
HLA-A*26:01
HLA-A*29:02
HLA-A*30:01
HLA-A*30:02
HLA-A*31:01
HLA-A*32:01
HLA-A*33:01
HLA-A*33:03
HLA-A*66:01
HLA-A*68:01
HLA-A*68:02
1
0.5
0
0.5
1
Protective
$-\log_{10} P$
Risk

(g)

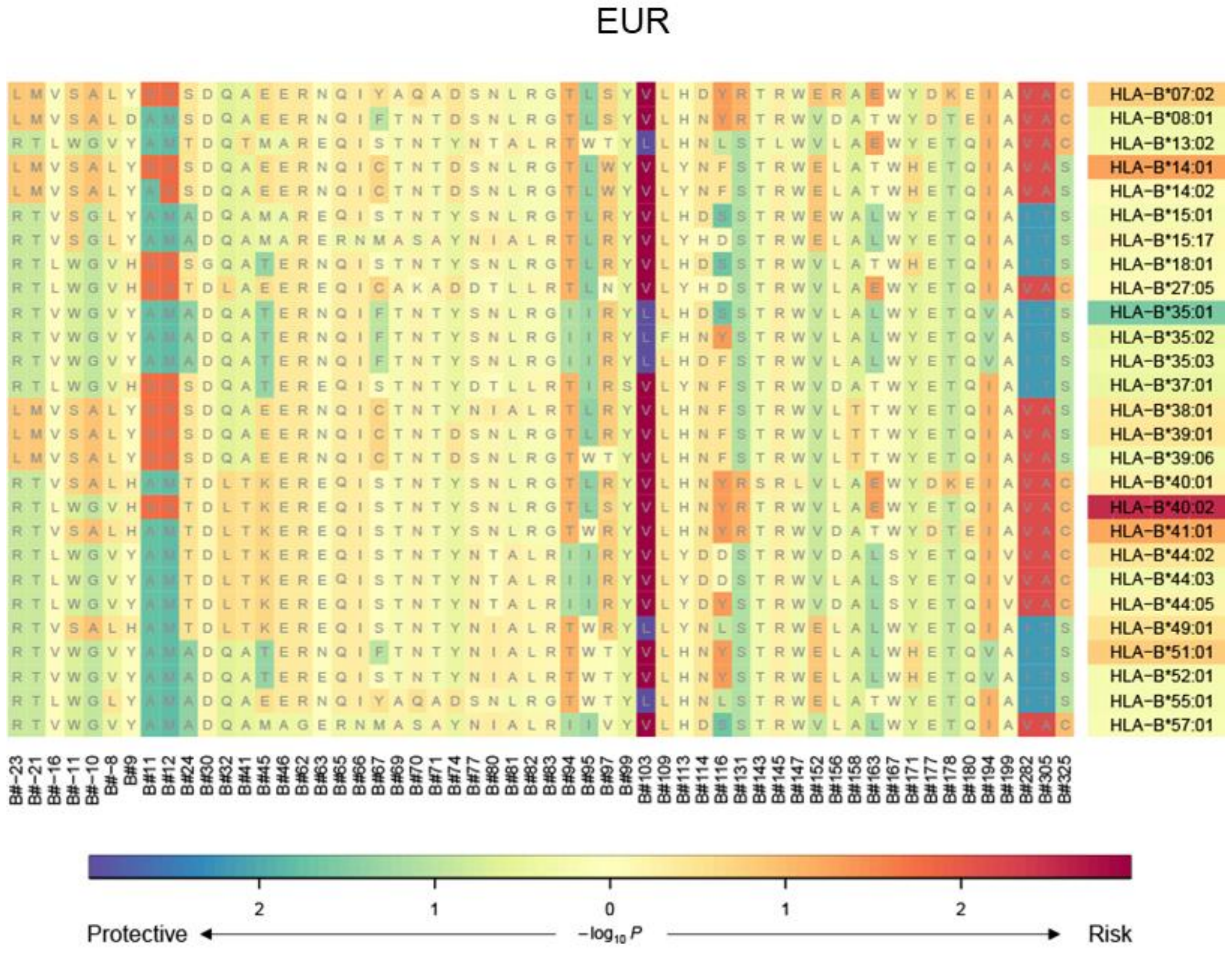
EUR
HLA-B*07:02
HLA-B*08:01
HLA-B*13:02
HLA-B*14:01
HLA-B*14:02
HLA-B*15:01
HLA-B*15:17
HLA-B*18:01
HLA-B*27:05
HLA-B*35:01
HLA-B*35:02
HLA-B*35:03
HLA-B*37:01
HLA-B*38:01
HLA-B*39:01
HLA-B*39:06
HLA-B*40:01
HLA-B*40:02
HLA-B*41:01
HLA-B*44:02
HLA-B*44:03
HLA-B*44:05
HLA-B*49:01
HLA-B*51:01
HLA-B*52:01
HLA-B*55:01
HLA-B*57:01
Protective
$-\log_{10} P$
Risk

(h)

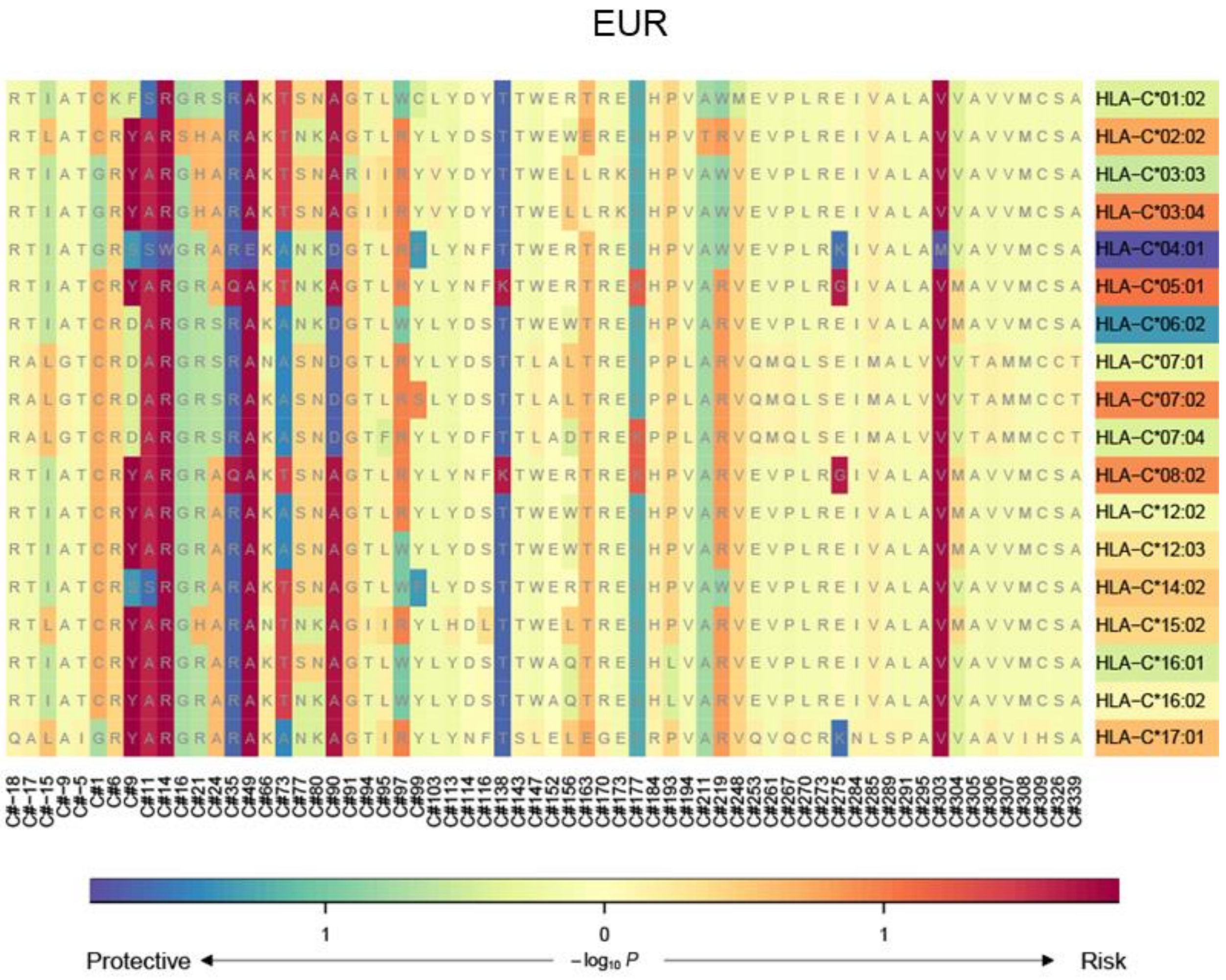
EUR
HLA-C*01:02
HLA-C*02:02
HLA-C*03:03
HLA-C*03:04
HLA-C*04:01
HLA-C*05:01
HLA-C*06:02
HLA-C*07:01
HLA-C*07:02
HLA-C*07:04
HLA-C*08:02
HLA-C*12:02
HLA-C*12:03
HLA-C*14:02
HLA-C*15:02
HLA-C*16:01
HLA-C*16:02
HLA-C*17:01
1
0
1
Protective
$-\log_{10} P$
Risk

(i)

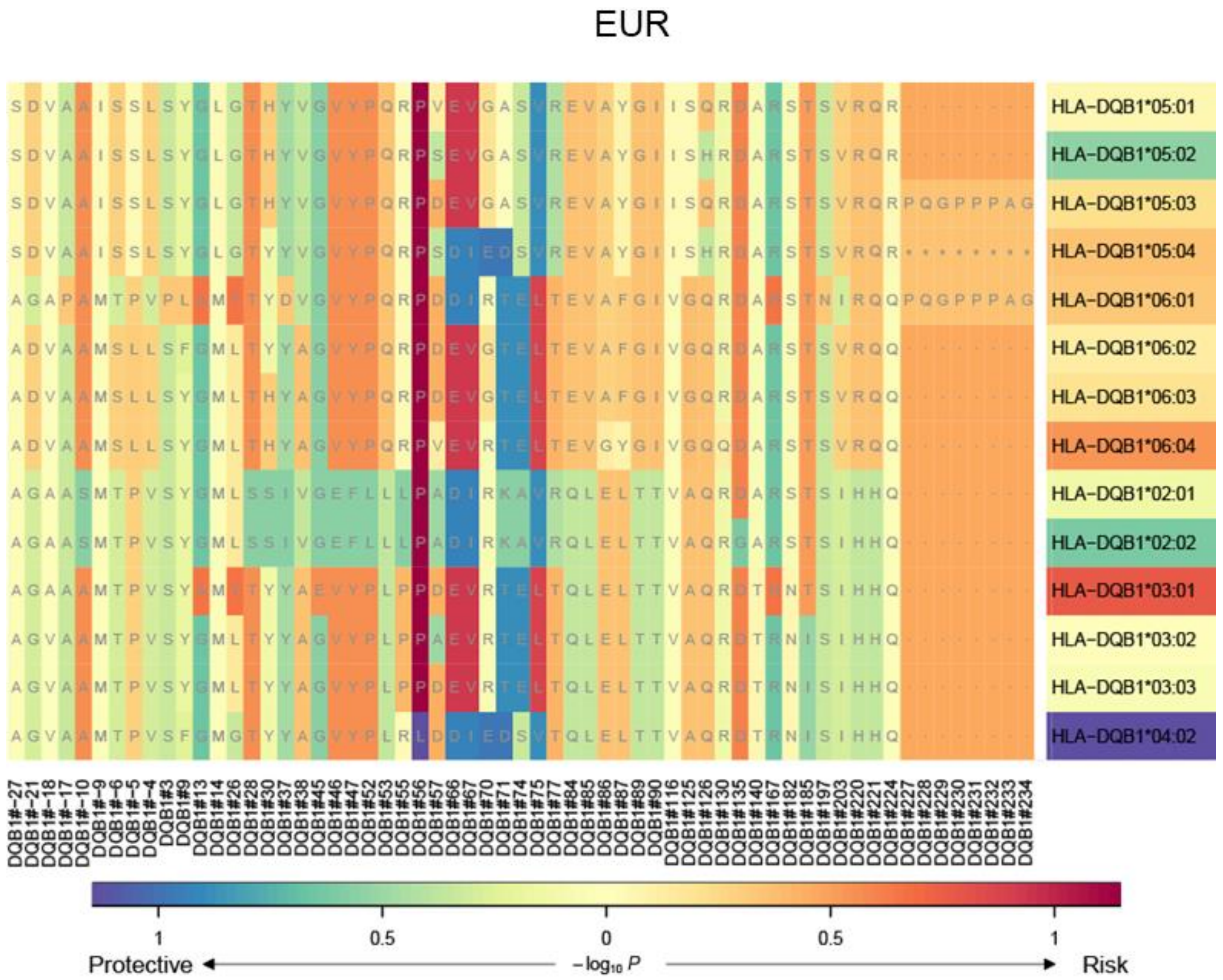

EUR
HLA-DQB1*05:01
HLA-DQB1*05:02
HLA-DQB1*05:03
HLA-DQB1*05:04
HLA-DQB1*06:01
HLA-DQB1*06:02
HLA-DQB1*06:03
HLA-DQB1*06:04
HLA-DQB1*02:01
HLA-DQB1*02:02
HLA-DQB1*03:01
HLA-DQB1*03:02
HLA-DQB1*03:03
HLA-DQB1*04:02
1
0.5
0
0.5
1
Protective
$-\log_{10} P$
Risk

(j)

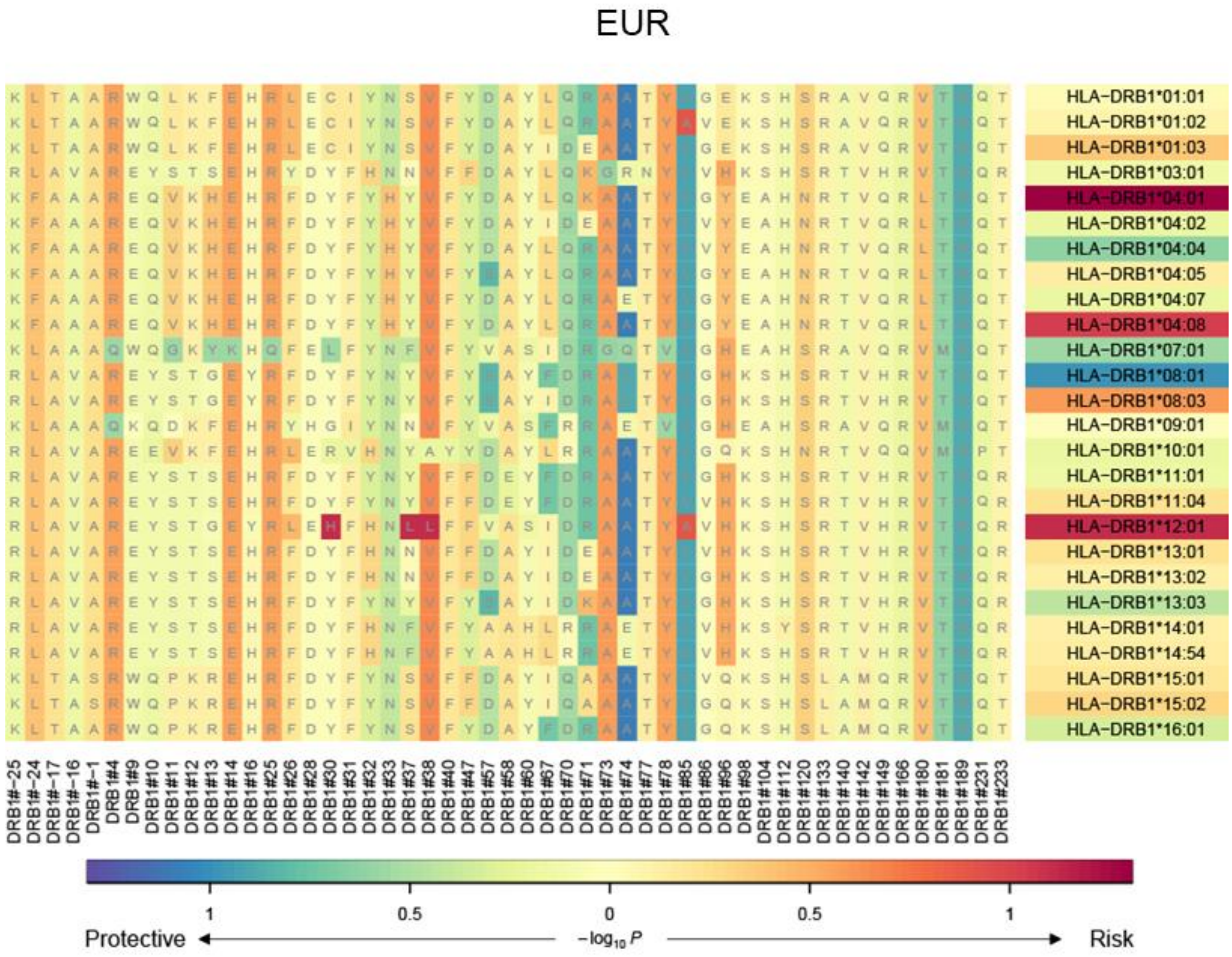

EUR
HLA-DRB1*01:01
HLA-DRB1*01:02
HLA-DRB1*01:03
HLA-DRB1*03:01
HLA-DRB1*04:01
HLA-DRB1*04:02
HLA-DRB1*04:04
HLA-DRB1*04:05
HLA-DRB1*04:07
HLA-DRB1*04:08
HLA-DRB1*07:01
HLA-DRB1*08:01
HLA-DRB1*08:03
HLA-DRB1*09:01
HLA-DRB1*10:01
HLA-DRB1*11:01
HLA-DRB1*11:04
HLA-DRB1*12:01
HLA-DRB1*13:01
HLA-DRB1*13:02
HLA-DRB1*13:03
HLA-DRB1*14:01
HLA-DRB1*14:54
HLA-DRB1*15:01
HLA-DRB1*15:02
HLA-DRB1*16:01
DRB1#-25
DRB1#-24
DRB1#-17
DRB1#-16
DRB1#-1
DRB1#4
DRB1#9
DRB1#10
DRB1#11
DRB1#12
DRB1#13
DRB1#14
DRB1#16
DRB1#25
DRB1#26
DRB1#28
DRB1#30
DRB1#31
DRB1#32
DRB1#33
DRB1#37
DRB1#38
DRB1#40
DRB1#47
DRB1#57
DRB1#58
DRB1#60
DRB1#67
DRB1#70
DRB1#71
DRB1#73
DRB1#74
DRB1#77
DRB1#78
DRB1#85
DRB1#86
DRB1#96
DRB1#98
DRB1#104
DRB1#112
DRB1#120
DRB1#133
DRB1#140
DRB1#142
DRB1#149
DRB1#166
DRB1#180
DRB1#181
DRB1#189
DRB1#231
DRB1#233
1
0.5
0
0.5
1
Protective
$-\log_{10} P$
Risk

(k)

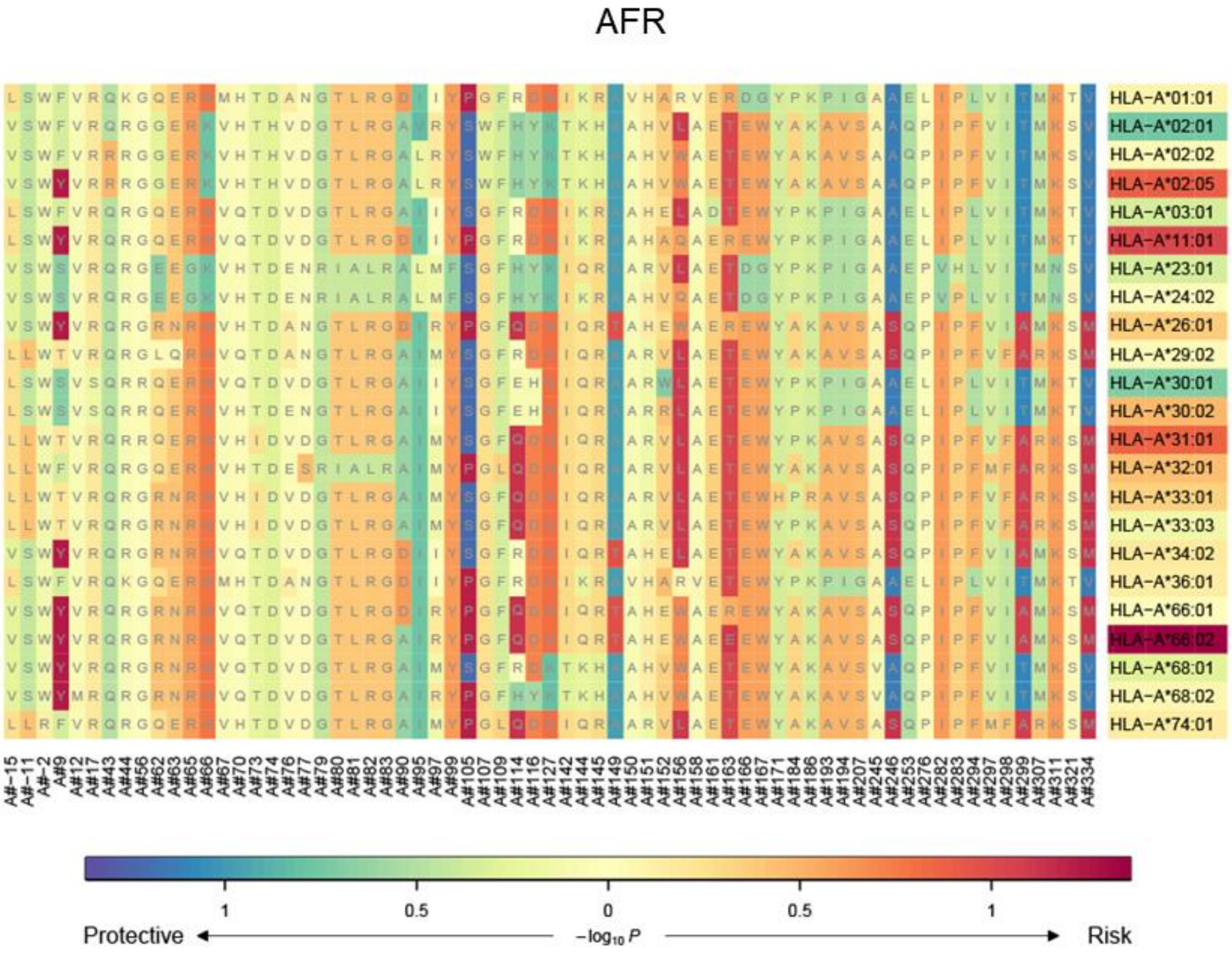
AFR
HLA-A*01:01
HLA-A*02:01
HLA-A*02:02
HLA-A*02:05
HLA-A*03:01
HLA-A*11:01
HLA-A*23:01
HLA-A*24:02
HLA-A*26:01
HLA-A*29:02
HLA-A*30:01
HLA-A*30:02
HLA-A*31:01
HLA-A*32:01
HLA-A*33:01
HLA-A*33:03
HLA-A*34:02
HLA-A*36:01
HLA-A*66:01
HLA-A*66:02
HLA-A*68:01
HLA-A*68:02
HLA-A*74:01
1
0.5
0
0.5
1
Protective
$-\log_{10} P$
Risk

(l)

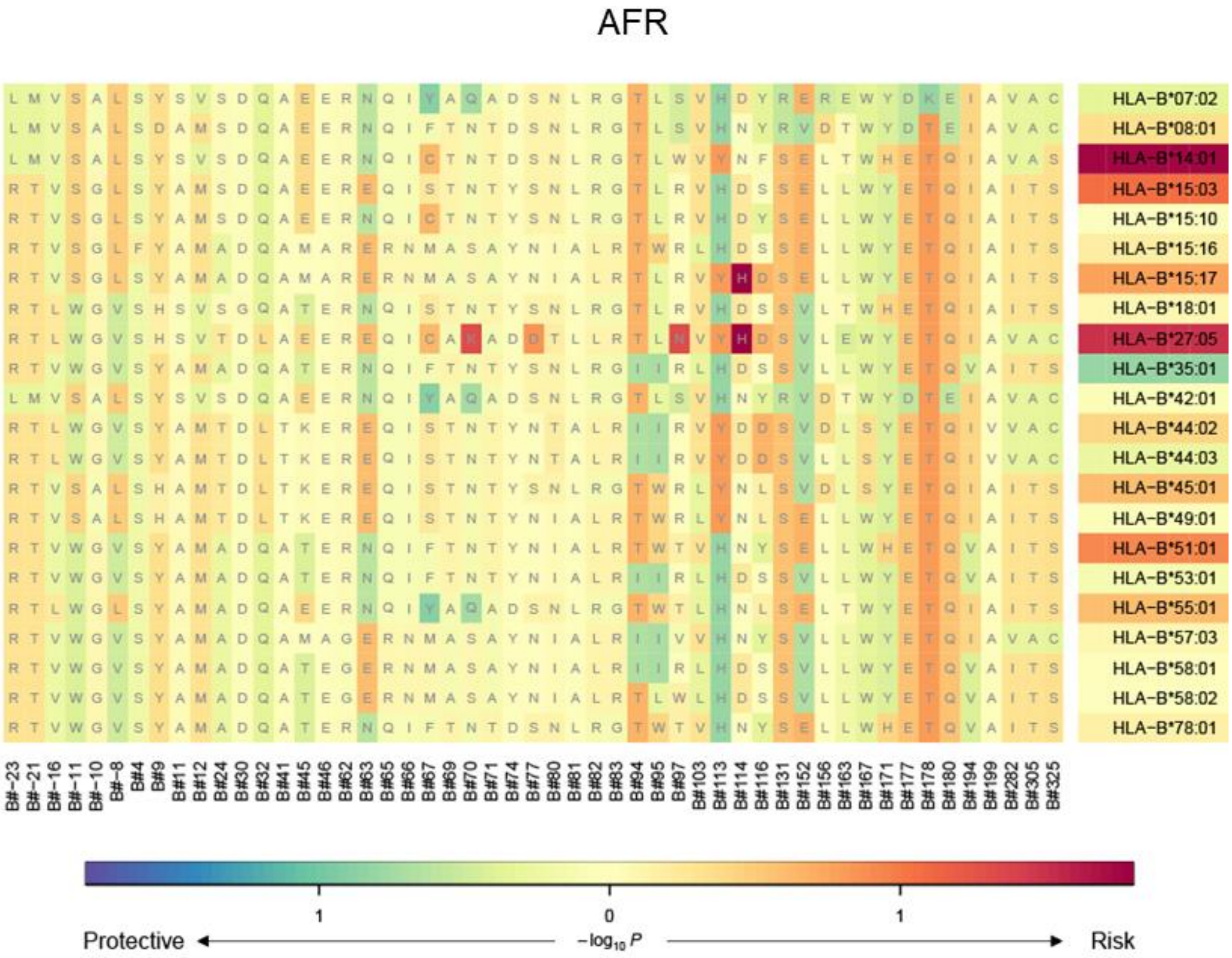
AFR
HLA-B*07:02
HLA-B*08:01
HLA-B*14:01
HLA-B*15:03
HLA-B*15:10
HLA-B*15:16
HLA-B*15:17
HLA-B*18:01
HLA-B*27:05
HLA-B*35:01
HLA-B*42:01
HLA-B*44:02
HLA-B*44:03
HLA-B*45:01
HLA-B*49:01
HLA-B*51:01
HLA-B*53:01
HLA-B*55:01
HLA-B*57:03
HLA-B*58:01
HLA-B*58:02
HLA-B*78:01
1
0
1
Protective
−log10 P
Risk

(m)

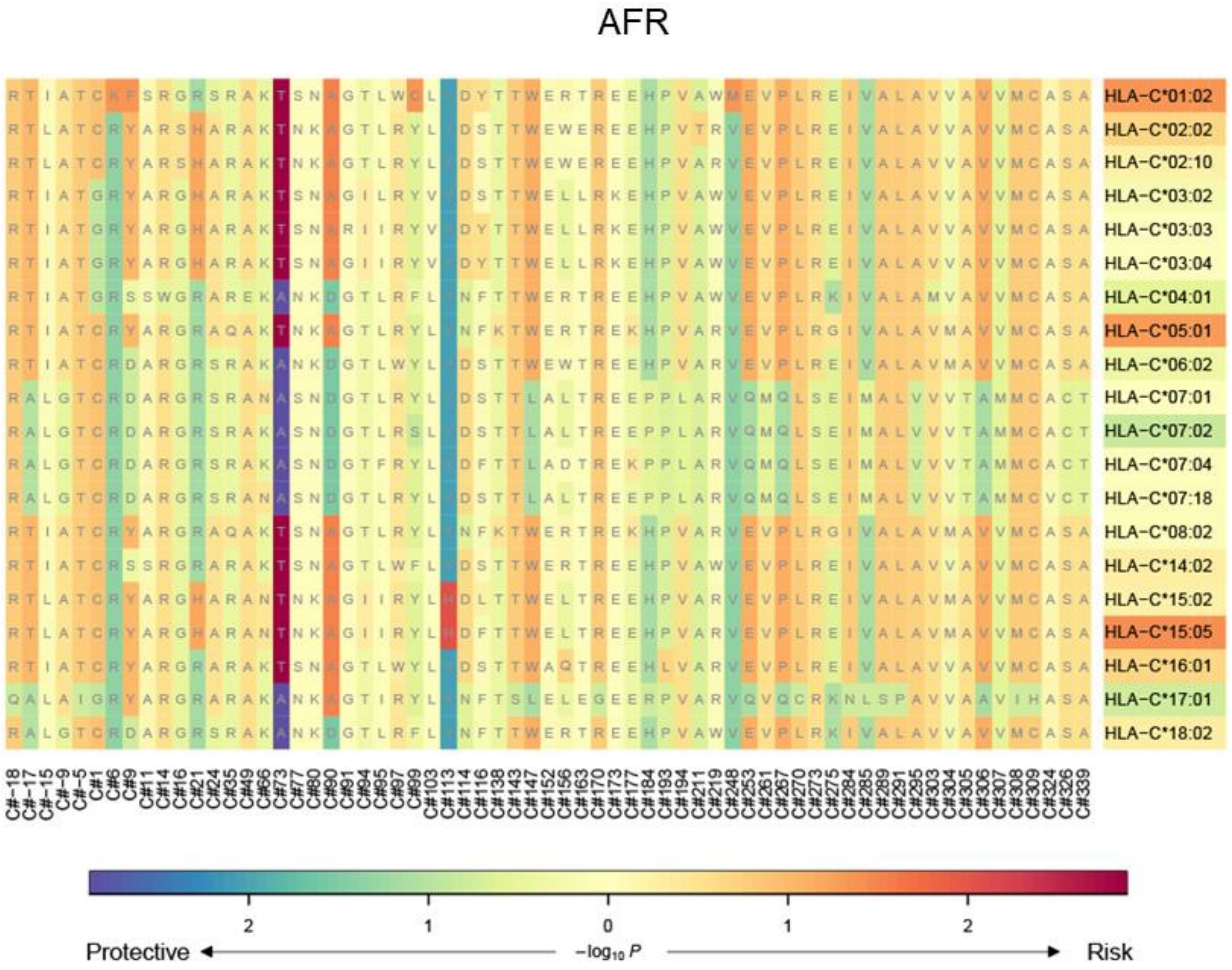
AFR
HLA-C*01:02
HLA-C*02:02
HLA-C*02:10
HLA-C*03:02
HLA-C*03:03
HLA-C*03:04
HLA-C*04:01
HLA-C*05:01
HLA-C*06:02
HLA-C*07:01
HLA-C*07:02
HLA-C*07:04
HLA-C*07:18
HLA-C*08:02
HLA-C*14:02
HLA-C*15:02
HLA-C*15:05
HLA-C*16:01
HLA-C*17:01
HLA-C*18:02
2
1
0
1
2
Protective
$-\log_{10} P$
Risk

(n)

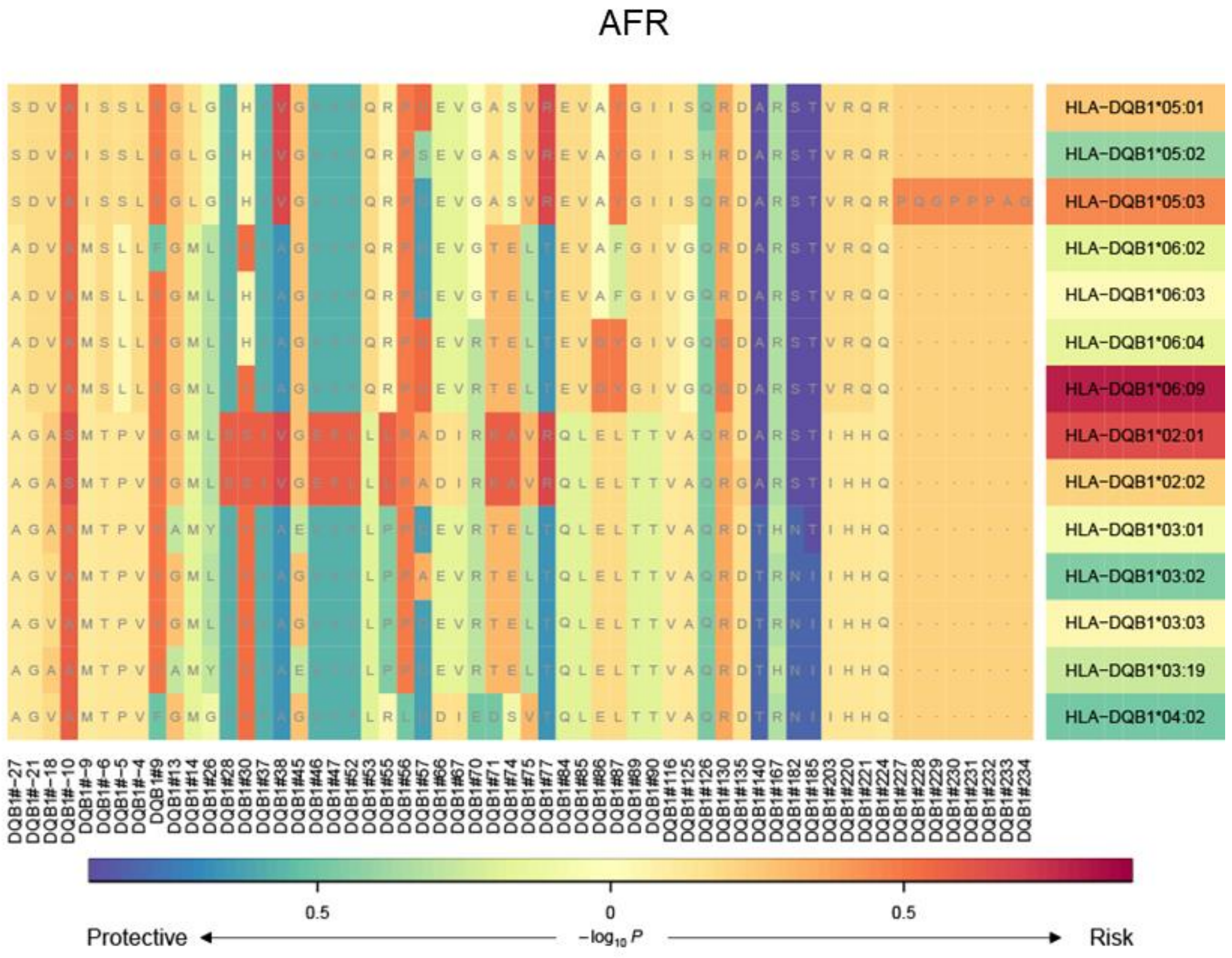
AFR
HLA-DQB1*05:01
HLA-DQB1*05:02
HLA-DQB1*05:03
HLA-DQB1*06:02
HLA-DQB1*06:03
HLA-DQB1*06:04
HLA-DQB1*06:09
HLA-DQB1*02:01
HLA-DQB1*02:02
HLA-DQB1*03:01
HLA-DQB1*03:02
HLA-DQB1*03:03
HLA-DQB1*03:19
HLA-DQB1*04:02
DQB1#-27
DQB1#-21
DQB1#-18
DQB1#-10
DQB1#-9
DQB1#-6
DQB1#-5
DQB1#-4
DQB1#9
DQB1#13
DQB1#14
DQB1#26
DQB1#28
DQB1#30
DQB1#37
DQB1#38
DQB1#45
DQB1#46
DQB1#47
DQB1#52
DQB1#53
DQB1#55
DQB1#56
DQB1#57
DQB1#66
DQB1#67
DQB1#70
DQB1#71
DQB1#74
DQB1#75
DQB1#77
DQB1#84
DQB1#85
DQB1#86
DQB1#87
DQB1#89
DQB1#90
DQB1#116
DQB1#125
DQB1#126
DQB1#130
DQB1#135
DQB1#140
DQB1#167
DQB1#182
DQB1#185
DQB1#203
DQB1#220
DQB1#221
DQB1#224
DQB1#227
DQB1#228
DQB1#229
DQB1#230
DQB1#231
DQB1#232
DQB1#233
DQB1#234
0.5
0
0.5
Protective
$-\log_{10} P$
Risk

(o)

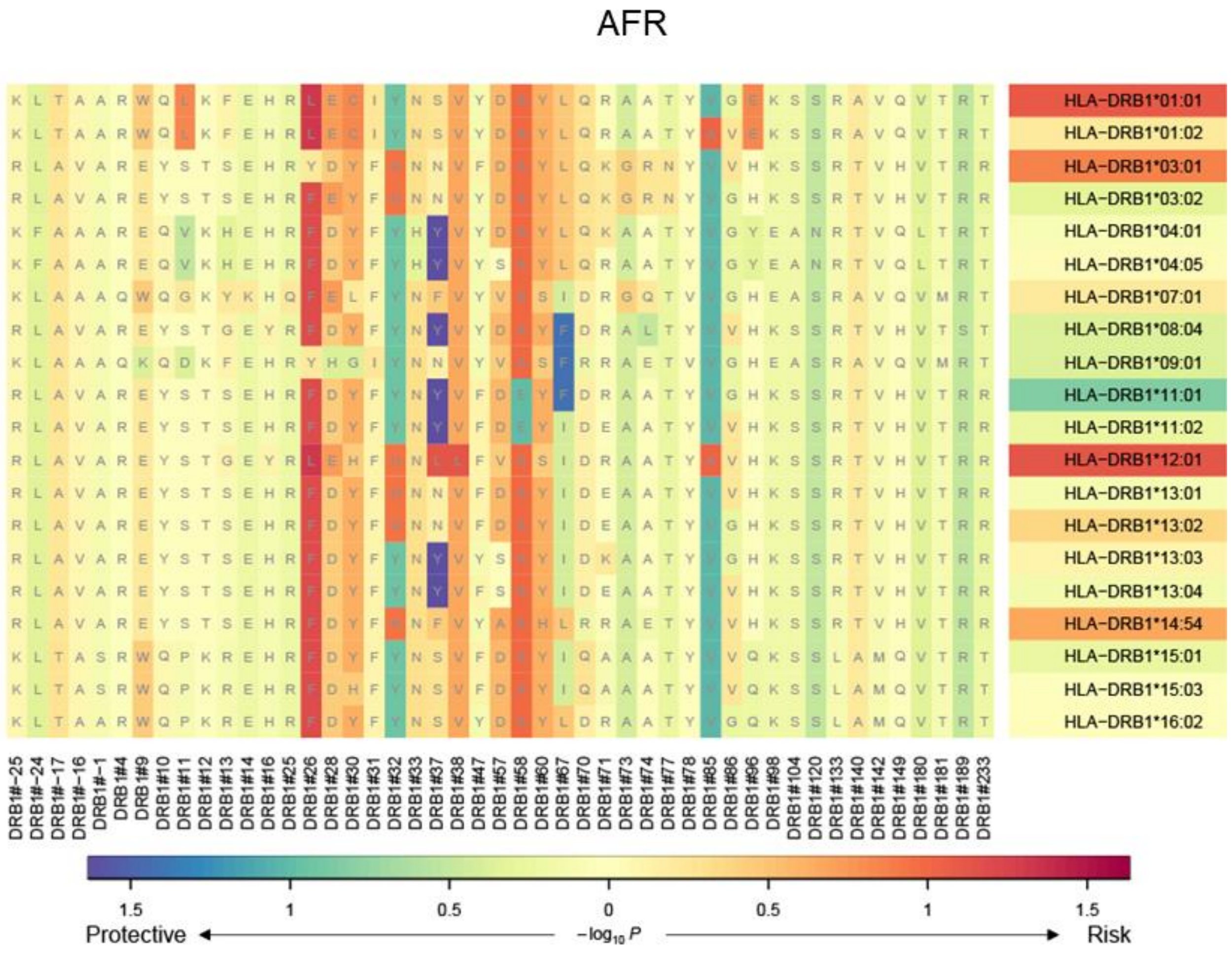

Supplementary Figure S4. Effects of individual residues at amino acid positions in HLA genes for pooled, European, and African ancestries. The color scheme represents the levels of effect and significance of the association between each amino acid residue at a given position for an HLA allele and late-onset asthma, adjusted for covariates. (a-e) HLA-A, HLA-B, HLA-C, HLA-DQB1, and HLA-DRB1 for pooled ancestries. (f-j) HLA-A, HLA-B, HLA-C, HLA-DQB1, and HLA-DRB1 for European ancestry. (k-o) HLA-A, HLA-B, HLA-C, HLA-DQB1, and HLA-DRB1 for African ancestry.

2.2 Supplementary Tables

Supplementary Table S1. Descriptive statistics for PEGS participants (n=3641)

	Asthma Cases	Asthma Controls	P-value
No. of participants	174	3467	
Age, mean ± SD (yr)	54.3 ± 11.1	50.3 ± 14.8	4.1×10^{-4}¶
Male, N (%)	27 (15.5)	1137 (32.8)	2.8×10^{-6}ǂ
Ancestry, mean ± SD (%)	5		
African	18.5 ± 32.8	12.6 ± 28.4	7.3×10^{-3}¶
Native American	1.4 ± 3.4	1.8 ± 6.4	0.4¶
East Asian	0.9 ± 1.6	2.7 ± 12.6	0.07¶
European	76.6 ± 33.0	79.9 ±31.5	0.2¶
Southeast Asian	2.4 ± 6.6	3.0 ± 9.2	0.4¶

¶We conducted unpaired two-sample t-tests for continuous variables.

ǂWe conducted two-proportion z-tests for categorical variables.

Supplementary Table S2. Results of fine-mapping of the MHC region harboring race/ethnicity-specific signals using sequenced data. We evaluated the joint effect of multiple variants on late-onset asthma using stepwise regression models. We conducted conditional analyses on race/ethnicity-specific lead SNPs to identify potential causal SNPs in the MHC region for all, White, and Black participants.

All Participants						
Variants	***Position***	***Gene (nearest)***	***Alleles***	***Freq?***	***OR [95% CI]***	***P-value***
rs9265901¶	6:31345615	HLA-B	A|G		1.01 [1.00, 1.03]	0.05
rs59377618	6:32820360	HLA-DOB	T|C		1.02 [1.01, 1.04]	4.42×10^{-3}
6:33097139	6:33097139	HLA-DPA1	G|A		0.98 [0.97, 0.99]	4.62×10^{-3}
rs2894334	6:33330567	SMIM40	A|C		0.99 [0.98, 0.99]	0.01
rs5009448	6:29972711	HLA-A	T|C		1.01 [1.00, 1.02]	0.02
rs7767589	6:30402196	RPP21	C|T		1.07 [1.02, 1.13]	7.00×10^{-3}
rs73728546	6:31380112	MICA	C|T		1.05 [1.02, 1.09]	5.24×10^{-3}
rs4569	6:31670030	LY6G5B	C|T		0.98 [0.97, 0.99]	3.21×10^{-3}
6:33061419	6:33061419	HLA-DPB1	C|T		1.10 [1.05, 1.16]	2.23×10^{-4}
rs9268541	6:32416750	BTNL2	T|C		1.03 [1.00, 1.05]	0.02
rs62407970	6:32969217	HLA-DMA	G|A		1.03 [1.01, 1.05]	6.89×10^{-3}
rs368207307	6:32524350	HLA-DRB5	C|G		1.02 [0.97, 1.08]	0.46
rs16871255	6:32965252	BRD2	T|C		1.02 [0.99, 1.06]	0.23
rs3135333	6:32996007	HLA-DOA	T|C		1.01 [0.99, 1.02]	0.12
rs13211714	6:33439483	ZBTB9	A|C		1.04 [0.99, 1.04]	0.11
rs2844624	6:31264470	HLA-C	T|C		0.99 [0.98, 1.00]	0.09
rs116062523	6:31383028	MICA	T|C		1.04 [0.99, 1.08]	0.08

**White Participants**						
rs55888430‡	6:32817365	HLA-DOB	G|A		1.03 [0.99, 1.07]	0.06
rs5009448	6:29972711	HLA-A	T|C		1.02 [1.01, 1.03]	1.65×10^{-3}
rs116062523	6:31383028	MICA	T|C		1.08 [1.03, 1.14]	3.64×10^{-3}
rs7754362	6:33143251	HCG24, COL11A2	A|C		0.98 [0.97, 0.99]	2.95×10^{-4}
6:29800571	6:29800571		A|C		1.07 [1.01, 1.13]	0.02
6:29816791	6:29816791		A|T		1.05 [1.00, 1.10]	0.04
6:31932537	6:31932537	CFB	T|C		1.07 [1.04, 1.11]	2.91×10^{-5}
rs1632859	6:31002121	MUC22	G|A		1.01 [1.00, 1.03]	0.04
rs17208209	6:32227599	NOTCH4	G|A		1.04 [1.00, 1.08]	0.04
rs115337486	6:33387741	KIFC1	C|T		1.04 [1.00, 1.08]	0.04
rs2040748	6:31276008	HLA-C	G|T		0.99 [0.98, 1.01]	0.73
rs116745041	6:32955914	BRD2	G|A		1.02 [0.97, 1.06]	0.45
rs3134772	6:31235440	HLA-C	G|C		1.01 [0.99, 1.02]	0.44
rs9258130	6:29710011	HLA-F	G|A		1.02 [0.98, 1.07]	0.25
rs2442753	6:31383956	MICA	T|C		0.99 [0.98, 1.01]	0.24
rs186455495	6:31311038	HLA-C	T|A		1.03 [0.99, 1.06]	0.07
rs183006581	6:30674381	DHX16	C|T		1.04 [0.99, 1.08]	0.06
rs9265866	6:31344981	HLA-B	C|T		1.02 [0.99, 1.03]	0.06
**Black Participants**						
rs117953947§	6:30244862	TRIM26, HCG17	G|A		1.13 [0.98, 1.30]	0.09

rs150891754	6:28608725	ZBED9	A|G		1.27 [1.13, 1.44]	$1.20{\times}10^{-4}$
rs17200414	6:31449359	MICB	C|T		1.21 [1.05, 1.38]	$7.90{\times}10^{-3}$
rs200535118	6:31352792	HLA-B	G|T		1.19 [1.01, 1.40]	0.04
rs62408569	6:29406052	OR5V1, OR12D2	C|T		1.31 [1.13, 1.52]	$4.04{\times}10^{-4}$
rs181714996	6:33331782	SMIM40	T|C		1.19 [1.07, 1.32]	$1.05{\times}10^{-3}$
rs115571466	6:32998090	HLA-DOA	A|G		1.20 [1.07, 1.35]	$1.71{\times}10^{-3}$
rs4713423	6:31034524	MUC22	G|C		1.04 [1.00, 1.08]	0.03
rs17198965	6:31308446	HLA-C	C|G		1.08 [1.02, 1.16]	0.01
rs73403122	6:32501344	HLA-DRB5	A|G		1.21 [1.07, 1.36]	$1.92{\times}10^{-3}$
rs17422797	6:32297751	NOTCH4	C|T		1.26 [1.09, 1.44]	$1.38{\times}10^{-3}$
rs79735834	6:29517075	MAS1L	A|G		1.08 [1.02, 1.15]	0.01
rs187569734	6:31044172	MUC22	G|A		1.28 [1.11, 1.48]	$6.82{\times}10^{-4}$
rs2245731	6:31262189	HLA-C	T|C		1.01 [0.97, 1.04]	0.70
rs3135356	6:32423739	HLA-DRA	G|A		1.03 [0.96, 1.10]	0.39
rs4569	6:31670030	LY6G5B	C|T		1.02 [0.99, 1.05]	0.25
rs114008864	6:30410517	RPP21	G|A		1.05 [0.98, 1.13]	0.17
rs3915970	6:31301683	HLA-C	T|C		1.04 [0.99, 1.09]	0.16
rs148696809	6:28966575	ZNF311	T|C		1.11 [0.99, 1.24]	0.06

¶Odds ratios represent the association between the lead SNPs (*rs9265901, rs55888430, rs117953947)* and late-onset asthma, adjusted for covariates and additional SNPs, in forward stepwise regression analyses. We adjusted all regression models for age, sex, and four ancestries.

Supplementary Table S3. Functional annotation of variants identified from fine-mapping analyses in the MHC region (chr6: 28,510,120-33,480,577). We gathered information on each variant's potential genomic regulatory role from publicly available data, including quantitative measures of functional importance (CADD score and RegDB rank and score), indication of functional genomic elements (ENCODE), and long-range chromatic interactions (Hi-C data from HUGIn) for all, White/Non-Hispanic, and Black/Non-Hispanic participants.

	All Participants							
**Variants**	**chr:BP (hg38)**	**CADD**	**RegDB¶**		**ENCODE**		**Hi-C (IMR90)**	
			Rank	***Score***	***Target of Assay***	***Bio-sample***	***IMR90***	***MES***
rs9265901	6:31345615	7.50	4	0.61			HCG27	HCG27
rs59377618	6:32820360	6.50	5	0.13			BTNL2	HLA-DMA
rs3130201	6:33097139	1.72	5	0.29			HLA-DPB2	HLA-DPB2
rs2894334	6:33330567	1.24	5	0.34			KIFC1	HLA-DPB2
rs5009448	6:29972711	5.41	4	0.61			HLA-G, HCG4	HLA-G, HCG4
rs7767589	6:30402196	2.72	5	0.13			TIM26	TRIM26
rs73728546	6:31380112	0.11	5	0.13	DNase, H3K4me3, H3K27ac, CTCF	Lung, B-cell, T-cell ($CD4^+$, $CD8^+$, helper), $CD14^+$		
rs4569	6:31670030	21.5	1f	0.55			MSH5; CLIC1	NEU1
rs566687684	6:33061419	0.76	7	0.18			HLA-DPB2	HLA-DPB2

rs9268541	6:32416750	0.12	5	0.59			HLA-DOB	HLA-DQB2
rs62407970	6:32969217	15.8	2b	0.82	CTCF	Lung, B-cell	HLA-DPB2	HLA-DPB2
rs368207307	6:32524350	11.5	5	0.65			HLA-DRB1 HLA-DRB6	
rs16871255	6:32965252	0.59	2b	0.01			HLA-DPB2	HLA-DPB2
rs3135333	6:32996007	1.92	5	0.17			HLA-DPB2	HLA-DPB2
rs13211714	6:33439483	13.1	4	0.61			PHF1<br>KIFC1	
rs2844624	6:31264470	6.67	6	0.49			PSORS1C1 CDSN C6orf15 PSORS1C2	PSORS1C1 CDSN C6orf15 PSORS1C2
rs116062523	6:31383028	11.7	4	0.61				
	**White Participants**							
rs55888430	6:32817365	1.07	4	0.61			BTNL2	BTNL2
rs5009448	6:29972711	5.41	4	0.61			HLA-G, HCG4	HLA-G, HCG4

rs116062523	6:31383028	11.7	4	0.61				
rs7754362	6:33143251	6.14	4	0.61	DNase, H3K4me3, H3K27ac, CTCF	IMR-90, lung, T-cell, B- cell, bronchial epithelial cell	HLA-DMA, BRD2	HLA-DMA, BRD2
rs1205233282	6:29800571	.	5	0.13				
rs41316358	6:29816791	.	5				HLA-J	HLA-J
rs529698255	6:31932537	2.54	7	0.18			MSH5	HLA-DOB
rs1632859	6:31002121	2.13	4	0.61			HCG22	HCG22
rs17208209	6:32227599	1.81	4	0.61			HLA-DOB	HLA-DOB
rs115337486	6:33387741	0.012	7	0.18			DAXX	
rs2040748	6:31276008	13.4	7	0.51				
rs116745041	6:32955914	1.02	4	0.61			HLA-DPB2	HLA-DPB2
rs3134772	6:31235440	4.02	3a	0.60			PSORS1C1 CDSN C6orf15 PSORS1C2	PSORS1C1 CDSN C6orf15 PSORS1C2

rs9258130	6:29710011	0.66	5	0.13				
rs2442753	6:31383956	5.77	5	0.18				
rs186455495	6:31311038	6.27	7	0.18				
rs183006581	6:30674381	0.03	7	0.18			TUBB MDC1 IER3 FLOT1	TUBB MDC1 IER3 FLOT1
rs9265866	6:31344981	7.33	4	0.61			HCG27	HCG27
	**Black Participants**							
rs117953947	6:30244862	0.69	3a	0.61			RPP21	RPP21
rs150891754	6:28608725	1.74	4	0.61				GPX5, GPX6
rs17200414	6:31449359	3.11	5	0.13			HLA-C	
rs200535118	6:31352792	0.18	3a	0.61				
rs62408569	6:29406052	8.47	5	0.13			ZNF311	LINC01015
rs181714996	6:33331782	1.07	5	1.0			KIFC1	
rs115571466	6:32998090	7.91	5	0.13			HLA-DPB2	HLA-DPB2
rs4713423	6:31034524	2.04	5	0.33			MUC22	MUC22
rs17198965	6:31308446	9.43	4	0.61	DNase	Colon, adrenal gland		

rs73403122	6:32501344	9.21	3a	0.96				HLA-DRB1, HLA-DRB6
rs17422797	6:32297751	6.24	5	0.54			HLA-DOB	HLA-DOB
rs79735834	6:29517075	2.77	4	0.61			MAS1L	MAS1L
rs187569734	6:31044172	0.83	3a	0.57			MUC22	MUC22
rs2245731	6:31262189	7.33	7	0.18			PSORS1C1 CDSN C6orf15 PSORS1C2	PSORS1C1 CDSN C6orf15 PSORS1C2
rs3135356	6:32423739	0.19	3a	0.85			HLA-DOB	HLA-DQB2
rs4569	6:31670030	21.5	1f	0.55			MSH5	NEU1
rs114008864	6:30410517	1.36	7	0.18			TRIM26	TRIM26
rs3915970	6:31301683	7.20	3a	0.70				
rs148696809	6:28966575	1.57	5	0.59				

¶The RegulomeDB score represents the likelihood that a variant is located in a functional region. The RegulomeDB variant classification scheme is defined in Boyle et al.[12]

Likely to affect binding

2b: TF binding + matched TF motif + matched DNase footprint + DNase peak

Minimal binding evidence

4: TF binding + DNase peak

5: TF binding or DNase peak

6: Motif hit

Supplementary Table S4. Full list of candidate loci for eQTLs. Some of the candidate causal variants identified from conditional analyses were weakly to strongly correlated with expression in multiple genes in lung tissue and whole blood according to the GTEx database.

All Participants

				Lung Tissue (GTEx)			***Whole Blood (GTEx)***		
SNP ID	*chr*	*Position (hg38)*	*Minor Allele*	*βeta*	*P-value*	*Gene*	*βeta*	*P-value*	*Gene*
rs9265901	6	31345615	G	-0.51	5.0×10^{-22}	*HLA-C*	-0.23	1.2×10^{-10}	*HLA-C*
rs9265901	6	31345615	G	0.52	1.7×10^{-11}	*HLA-S*	0.53	1.4×10^{-15}	*HLA-S*
rs59377618	6	32820360	C	-0.26	1.4×10^{-4}	*HLA-DOB*	-0.24	5.6×10^{-10}	*HLA-DOB*
rs3130201	6	33097139	A	0.49	2.6×10^{-18}	*HLA-DPB2*	0.44	2.1×10^{-28}	*HLA-DPB2*
rs3130201	6	33097139	A	-0.17	5.2×10^{-6}	*RPL32P1*	-0.14	1.2×10^{-6}	*RPL32P1*
rs2894334	6	33330567	C	-0.13	6.3×10^{-7}	*DAXX*	-0.14	2.1×10^{-11}	*DAXX*

rs2894334	6	33330567	C	0.10	5.0×10^{-6}	*ZBTB22*	0.13	4.0×10^{-9}	*ZBTB22*
rs2894334	6	33330567	C				-0.17	2.8×10^{-9}	*BAK1*
rs2894334	6	33330567	C				0.06	1.3×10^{-6}	*RPS18*
rs2894334	6	33330567	C				0.1	2.9×10^{-14}	*TAPBP*
rs5009448	6	29972711	C	0.5	6.7×10^{-18}	HCP5B	0.45	6.8×10^{-18}	HLA-H
rs5009448	6	29972711	C	0.44	1.1×10^{-11}	MICD	0.46	2.2×10^{-17}	HCP5B
rs5009448	6	29972711	C	0.42	1.5×10^{-11}	HLA-H	0.33	1.1×10^{-12}	HCG9
rs5009448	6	29972711	C	-0.15	6.6×10^{-8}	ZNRD1ASP	0.30	3.7×10^{-9}	HLA-J
rs5009448	6	29972711	C	-0.28	1.6×10^{-6}	HCG4P7	0.28	8.2×10^{-9}	MICD
rs5009448	6	29972711	C	-0.29	1.2×10^{-5}	HCG17	0.26	2.8×10^{-7}	DDX39BP2
rs5009448	6	29972711	C	0.27	7.2×10^{-5}	ZFP57	0.28	5.0×10^{-7}	ZFP57
rs5009448	6	29972711	C	0.19	8.1×10^{-5}	RPL23AP1	0.22	1.2×10^{-6}	HCG4P3
rs5009448	6	29972711	C	0.16	3.1×10^{-4}	HCG4P3	0.18	4.3×10^{-6}	HLA-F-AS1
rs5009448	6	29972711	C				0.24	5.9×10^{-6}	HLA-K
rs5009448	6	29972711	C				-0.21	5.9×10^{-6}	HCG4P7
rs5009448	6	29972711	C				0.07	1.8×10^{-5}	PPP1R11
rs5009448	6	29972711	C				0.10	2.6×10^{-5}	GABBR1
rs5009448	6	29972711	C				0.19	1.8×10^{-4}	RPL23AP1

rs4569	6	31670030	T	-0.53	1.8×10^{-43}	LY6G5C	-0.74	1.3×10^{-119}	LY6G5C
rs4569	6	31670030	T	-0.22	1.2×10^{-29}	LY6G5B	-0.23	1.8×10^{-33}	LY6G5B
rs4569	6	31670030	T	-0.28	1.5×10^{-6}	HLA-DRB5	-0.09	9.9×10^{-9}	AIF1
rs4569	6	31670030	T	0.29	3.9×10^{-6}	STK19B	-0.07	2.4×10^{-7}	PRRC2A
rs4569	6	31670030	T	-0.11	9.0×10^{-6}	DDAH2	0.26	3.5×10^{-6}	STK19B
rs4569	6	31670030	T	-0.12	1.2×10^{-4}	ABHD16A	-0.18	2.6×10^{-5}	LY6G6D
rs4569	6	31670030	T	-0.11	1.5×10^{-4}	ATF6B	0.09	3.1×10^{-5}	HCG27
rs4569	6	31670030	T	0.14	2.5×10^{-4}	HCG27	-0.16	5.0×10^{-5}	LINC00243
rs4569	6	31670030	T				-0.16	5.1×10^{-5}	HLA-DRB5
rs4569	6	31670030	T				-0.17	2.3×10^{-4}	VWA7
rs9268541	6	32416750	C	0.45	2.9×10^{-5}	*HLA-DRB9*	-0.27	3.4×10^{-5}	*HLA-DRB5*

White Participants

				Lung Tissue (GTEx)			Whole Blood (GTEx)		
SNP ID	*chr*	*Position (hg38)*	*Minor Allele*	*βeta*	*P-value*	*Gene*	*βeta*	*P-value*	*Gene*
rs116062523	6	31383028	C				0.69	2.5×10^{-5}	MICB
rs7754362	6	33143251	C	-0.11	4.2×10^{-5}	RPS18			
rs1632859	6	31002121	A	-0.34	3.2×10^{-7}	PSORS1C3	0.28	5.5×10^{-7}	HLA-S
rs1632859	6	31002121	A	0.33	1.4×10^{-6}	HLA-S	0.14	2.3×10^{-6}	HLA-C
rs1632859	6	31002121	A	-0.18	5.1×10^{-5}	C4A	0.14	4.0×10^{-6}	CCHCR1
rs1632859	6	31002121	A	-0.23	6.5×10^{-5}	POU5F1	0.21	4.6×10^{-5}	HLA-J
rs1632859	6	31002121	A	0.14	1.0×10^{-4}	CCHCR1	-0.14	2.3×10^{-4}	PSORS1C3
rs1632859	6	31002121	A	0.2	1.2×10^{-4}	HLA-J			
rs1632859	6	31002121	A	0.14	2.7×10^{-4}	HCG27			

rs115337486	6	33387741	T	-0.34	1.0×10^{-4}	DAXX			

Black Participants

				Lung Tissue (GTEx)			Whole Blood (GTEx)		
SNP ID	*chr*	*Position (hg38)*	*Minor Allele*	*βeta*	*P-value*	*Gene*	*βeta*	*P-value*	*Gene*
rs117953947	6	30244862	A	-0.41	5.1×10^{-5}	ZNRD1ASP			
rs17200414	6	31449359	T	-0.43	1.5×10^{-8}	MICA	-0.27	3.4×10^{-4}	MICA
rs200535118	6	31352792	T	-0.55	2.1×10^{-4}	*POU5F1*	0.59	6.0×10^{-6}	*MIR6891*
rs62408569	6	29406052	T	0.61	8.8×10^{-7}	MICE	0.56	3.6×10^{-4}	*HLA-U*
rs62408569	6	29406052	T	-0.49	3.0×10^{-6}	OR2H2			
rs62408569	6	29406052	T	0.71	2.3×10^{-5}	HLA-U			
rs4713423	6	31034524	C	0.69	5.9×10^{-24}	PSORS1C3	0.37	4.9×10^{-19}	PSORS1C3
rs4713423	6	31034524	C	0.44	4.6×10^{-12}	HCG22	-0.25	5.6×10^{-14}	HLA-C

rs4713423	6	31034524	C	-0.33	3.2×10^{-11}	HLA-C	0.11	4.8×10^{-7}	HCG27
rs4713423	6	31034524	C	0.27	3.5×10^{-11}	HCG27	0.26	1.3×10^{-6}	CYP21A1P
rs4713423	6	31034524	C	0.28	3.8×10^{-6}	POU5F1	0.16	6.5×10^{-6}	POU5F1
rs4713423	6	31034524	C				0.11	1.5×10^{-4}	HCG18
rs17198965	6	31308446	G	-0.5	7.5×10^{-5}	POU5F1			
rs73403122	6	32501344	G	0.51	8.4×10^{-5}	HLA-DOA			
rs73403122	6	32501344	G	0.81	9.7×10^{-5}	HLA-DRB9			
rs17422797	6	32297751	T	-0.46	2.8×10^{-5}	HLA-DQB2	-0.36	5.2×10^{-5}	HLA-DQB2
rs17422797	6	32297751	T	0.44	3.6×10^{-5}	HCG23			
rs17422797	6	32297751	T	0.23	9.0×10^{-5}	TAP2			
rs17422797	6	32297751	T	0.46	1.6×10^{-4}	STK19B			

Supplementary Table S5. Functional annotation of variants in the MHC region identified from fine-mapping analyses (chr6: 28,510,120-33,480,577). We gathered information on each variant's potential genomic regulatory role from publicly available data, including quantitative measures of functional importance (CADD score and RegDB rank and score), indication of functional genomic elements (ENCODE), and long-range chromatic interactions (Hi-C data from HUGIn) for all, White, and Black participants.

	All Participants							
**Variants**	**chr:BP (hg38)**	**CADD**	**RegDB**[¶]		**ENCODE**		**Hi-C**	
			Rank	***Score***	***Target of Assay***	***Bio-sample***	***IMR90***	***MES***
rs9265901[¶]	6:31345615	7.50	4	0.61			HCG27	HCG27
rs59377618	6:32820360	6.50	5	0.13			BTNL2	HLA-DMA
rs3130201	6:33097139	1.72	5	0.29			HLA-DPB2	HLA-DPB2
rs2894334	6:33330567	1.24	5	0.34			KIFC1	HLA-DPB2
rs5009448	6:29972711	5.41	4	0.61			HLA-G, HCG4	HLA-G, HCG4
rs7767589	6:30402196	2.72	5	0.13			TIM26	TRIM26
rs73728546	6:31380112	0.11	5	0.13	DNase	Lung, CD8+ cell line		
rs4569	6:31670030	21.5	1f	0.55			MSH5; CLIC1	NEU1
rs566687684	6:33061419	0.76	7	0.18			HLA-DPB2	HLA-DPB2
rs9268541	6:32416750	0.12	5	0.59			HLA-DOB	HLA-DQB2

rs62407970	6:32969217	15.8	2b	0.82	DNase, H3K4me3, H3K27ac	Lung, CD14+, CD8+, CD4+, naïve B, natural killer cell lines	HLA-DPB2	HLA-DPB2
	***White* Participants**							
rs55888430‡	6:32817365	1.07	4	0.61			BTNL2	BTNL2
rs5009448	6:29972711	5.41	4	0.61			HLA-G, HCG4	HLA-G, HCG4
rs116062523	6:31383028	11.7	4	0.61				
rs7754362	6:33143251	6.14	4	0.61	DNase, CTCF	Lung, CD14+, CD4+, CD8+, IMR90 cell lines	HLA-DMA, BRD2	HLA-DMA, BRD2
rs1205233282	6:29800571	.	5	0.13				
rs41316358	6:29816791	.	5				HLA-J	HLA-J
rs529698255	6:31932537	2.54	7	0.18			MSH5	HLA-DOB
rs1632859	6:31002121	2.13	4	0.61			HCG22	HCG22
rs17208209	6:32227599	1.81	4	0.61			HLA-DOB	HLA-DOB
rs115337486	6:33387741	0.012	7	0.18			DAXX	
	***Black* Participants**							
rs117953947§	6:30244862	0.69	3a	0.61			RPP21	RPP21

rs150891754	6:28608725	1.74	4	0.61				GPX5, GPX6
rs17200414	6:31449359	3.11	5	0.13			HLA-C	
rs200535118	6:31352792	0.18	3a	0.61				
rs62408569	6:29406052	8.47	5	0.13			ZNF311	LINC01015
rs181714996	6:33331782	1.07	5	1.0			KIFC1	
rs115571466	6:32998090	7.91	5	0.13			HLA-DPB2	HLA-DPB2
rs4713423	6:31034524	2.04	5	0.33			MUC22	MUC22
rs17198965	6:31308446	9.43	4	0.61	DNase, H3K4me3	Lung, CD14+, CD4+, CD8+, B, bronchial epithelial, T helper 17 cell lines		
rs73403122	6:32501344	9.21	3a	0.96				HLA-DRB1, HLA-DRB6
rs17422797	6:32297751	6.24	5	0.54			HLA-DOB	HLA-DOB
rs79735834	6:29517075	2.77	4	0.61			MAS1L	MAS1L
rs187569734	6:31044172	0.83	3a	0.57			MUC22	MUC22

¶The RegulomeDB (RegDB) score represents the likelihood that a variant is located in a functional region. The RegDB variant classification scheme is defined in Boyle et al. (2012).

Likely to affect binding

2b: TF binding + matched TF motif + matched DNase footprint + DNase peak

Minimal binding evidence

4: TF binding + DNase peak

5: TF binding or DNase peak

6: Motif hit

3 References

Akhtari, F.S., Lloyd, D., Burkholder, A., Tong, X., House, J.S., Lee, E.Y., et al. (2023). Questionnaire-Based Polyexposure Assessment Outperforms Polygenic Scores for Classification of Type 2 Diabetes in a Multiancestry Cohort. *Diabetes Care* 46(1-9). doi: 10.2337/dc22-0295.

Boyle, A.P., Hong, E.L., Hariharan, M., Cheng, Y., Schaub, M.A., Kasowski, M., et al. (2012). Annotation of functional variation in personal genomes using RegulomeDB. *Genome Res* 22(9)**,** 1790-1797.

Byrska-Bishop, M., Evani, U.S., Zhao, X., Basile, A.O., Abel, H.J., Regier, A.A., et al. (2022). High-coverage whole-genome sequencing of the expanded 1000 Genomes Project cohort including 602 trios. *Cell* 185(18)**,** 3426-3440.e3419. doi: 10.1016/j.cell.2022.08.004.

ENCODE Project Consortium (2012). An integrated encyclopedia of DNA elements in the human genome. *Nature* 489(7414)**,** 57-74. doi: 10.1038/nature11247.

Fairley, S., Lowy-Gallego, E., Perry, E., and Flicek, P. (2020). The International Genome Sample Resource (IGSR) collection of open human genomic variation resources. *Nucleic Acids Res* 48(D1)**,** D941-d947. doi: 10.1093/nar/gkz836.

Genotype-Tissue Expression (GTEx) Project (2022). GTEx Portal.

Ghoussaini, M., Mountjoy, E., Carmona, M., Peat, G., Schmidt, E.M., Hercules, A., et al. (2021). Open Targets Genetics: systematic identification of trait-associated genes using large-scale genetics and functional genomics. *Nucleic Acids Res* 49(D1)**,** D1311-d1320. doi: 10.1093/nar/gkaa840.

Lee, E.Y., Mak, A.C.Y., Hu, D., Sajuthi, S., White, M.J., Keys, K.L., et al. (2020). Whole-genome sequencing identifies novel functional loci associated with lung function in Puerto Rican youth. *Am J Respir Crit Care Med* 202(7)**,** 962-972. doi: 10.1164/rccm.202002-0351OC.

Lee, H., and Kingsford, C. (2018). Kourami: graph-guided assembly for novel human leukocyte antigen allele discovery. *Genome Biol* 19(1), 16. doi: 10.1186/s13059-018-1388-2.
Li, H., and Durbin, R. (2010). Fast and accurate long-read alignment with Burrows-Wheeler transform. *Bioinformatics* 26(5), 589-595. doi: 10.1093/bioinformatics/btp698.
Luo, Y., Hitz, B.C., Gabdank, I., Hilton, J.A., Kagda, M.S., Lam, B., et al. (2020). New developments on the Encyclopedia of DNA Elements (ENCODE) data portal. *Nucleic Acids Res* 48(D1), D882-d889. doi: 10.1093/nar/gkz1062.
Maples, B.K., Gravel, S., Kenny, E.E., and Bustamante, C.D. (2013). RFMix: a discriminative modeling approach for rapid and robust local-ancestry inference. *Am J Hum Genet* 93(2), 278-288. doi: 10.1016/j.ajhg.2013.06.020.
Martin, J.S., Xu, Z., Reiner, A.P., Mohlke, K.L., Sullivan, P., Ren, B., et al. (2017). HUGIn: Hi-C Unifying Genomic Interrogator. *Bioinformatics* 33(23), 3793-3795. doi: 10.1093/bioinformatics/btx359.
Mountjoy, E., Schmidt, E.M., Carmona, M., Schwartzentruber, J., Peat, G., Miranda, A., et al. (2021). An open approach to systematically prioritize causal variants and genes at all published human GWAS trait-associated loci. *Nat Genet* 53(11), 1527-1533. doi: 10.1038/s41588-021-00945-5.
Purcell, S., Neale, B., Todd-Brown, K., Thomas, L., Ferreira, M.A., Bender, D., et al. (2007). PLINK: a tool set for whole-genome association and population-based linkage analyses. *Am J Hum Genet* 81(3), 559-575. doi: 10.1086/519795.
Robinson, J., Barker, D.J., Georgiou, X., Cooper, M.A., Flicek, P., and Marsh, S.G.E. (2020). IPD-IMGT/HLA Database. *Nucleic Acids Res* 48(D1), D948-d955. doi: 10.1093/nar/gkz950.
